# Nano Si‐Doped Ruthenium Oxide Particles from Caged Precursors for High‐Performance Acidic Oxygen Evolution

**DOI:** 10.1002/advs.202207429

**Published:** 2023-02-21

**Authors:** Chunxiang Liu, Yunbo Jiang, Teng Wang, Qiaosheng Li, Yuzhou Liu

**Affiliations:** ^1^ School of Chemistry Beihang University Beijing 100191 P. R. China; ^2^ State Key Laboratory for Advanced Technologies for Comprehensive Utilization of Platinum Metals Sino‐Platinum Metals Co. Ltd. 650221 Kunming P. R. China; ^3^ Beijing Advanced Innovation Center for Biomedical Engineering School of Chemistry Beihang University Beijing 100191 P. R. China; ^4^ Research Department Shenyunzhihe Company Qidian Center, FL 10, Energy East Rd, #1, Changping District Beijing 102206 P. R. China

**Keywords:** caged precursors, oxygen evolution reaction, ruthenium oxide particles

## Abstract

RuO_2_ is well known as the benchmark acidic oxygen evolution reaction (OER) catalyst, but its practical application has been impeded by its limited durability. Herein, it is presented that the stability of ruthenium oxide can be significantly improved by pretrapping RuCl_3_ precursors within a cage compound possessing 72 aromatic rings, which leads to well carbon‐coated RuO_*x*_ particles (Si‐RuO_*x*_@C) after calcination. The catalyst survives in 0.5 M H_2_SO_4_ for an unprecedented period of 100 hours at 10 mA cm^−2^ with minimal overpotential change during OER. In contrast, RuO_*x*_ prepared from similar non‐tied compounds doesn't exhibit such catalytic activity, highlighting the importance of the preorganization of Ru precursors within the cage prior to calcination. In addition, the overpotential at 10 mA cm^−2^ in acid solution is only 220 mV, much less than that of commercial RuO_2_. X‐ray absorption fine structure (FT‐EXAFS) reveals the Si doping through unusual Ru–Si bond, and density functional theory (DFT) calculation reveals the importance of the Ru‐Si bond in enhancing both the activity and stability of the catalyst.

## Graphical abstract

Unique Si‐RuO_*x*_@C from caged precursors has excellent OER catalytic activity and high stability in acidic working conditions, and could maintain 100 h stability in 0.5 M H_2_SO_4_ with the working current of 20 mA cm^−^^2^.

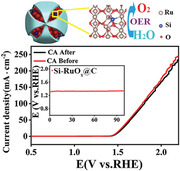

## 1 Introduction

In recent decades, the problem of energy crisis and environmental pollution has been increasingly prominent, and clean energy source from water splitting has been considered as a promising way to solve this issue. However, the oxygen evolution reaction (OER) for water splitting is a 4‐electron transfer process which is kinetically slow^[^ ^1^, ^2^, ^3^, ^4^, ^5^, ^6^ ^]^ in addition to the high thermodynamic potential of 1.23 V (vs. RHE).^[^ ^7^, ^8^, ^9^ ^]^ Volumes of work has been devoted to the discovery of efficient and stable OER catalysts. Although great progress has been achieved in alkaline electrolytes with abundant transition metal compounds,^[^ ^10^, ^11^, ^12^, ^13^, ^14^, ^15^, ^16^, ^17^ ^]^ the need of high current density in practical water‐splitting devices drives the search of suitable catalysts for acidic OER.

Iridium oxide has been commercially used as acidic OER catalyst in water splitting due to its high durability, but it suffers from high overpotentials, while ruthenium oxide can produce oxygen with much lower overpotentials, but it is readily oxidized to soluble RuO_4_ at low pHs and thus shortly loses activity.^[^ ^18^, ^19^, ^20^, ^21^, ^22^, ^23^, ^24^, ^25^, ^26^, ^27^, ^28^, ^29^, ^30^ ^]^ Various approaches have been proposed to increase the oxidation stability of ruthenium ion.^[^ ^1^, ^31^, ^32^ ^]^ Specifically carbon coating and heteroatom doping are effective for this purpose.^[^ ^33^, ^34^, ^35^ ^]^ For example, it is shown by Deng et al. that the graphene coating on the Ru‐Ni center surface acted as an electron donor that enhanced the stability up to 24 h. In these cases, the molecular‐level interaction between graphene and metal particles effectively boosted stability.^[^ ^34^ ^]^ Based on these achievements, we reasoned that preorganization of molecular species of carbon sources with metal ions before calcination could further elevate the effectiveness of this approach, therefore generating more stable OER catalysts.

Recently we developed a facile synthesis of an organic cage (COP1‐T) with the diameters of several nanometers,^[^ ^36^ ^]^ and subsequently show effectiveness of trapping various metal ions through coordinating carboxylate groups on its surface.^[^ ^37^, ^38^ ^]^ It was also demonstrated that unique selectivity could be obtained using these assemblies as catalysts for several kinds of reaction. Considering the suitability as carbon source for COP1‐T with 72 phenyl rings per cage, we decided to evaluate the possibility of preorganizing ruthenium ions within a cage followed by calcination for a stable carbon‐coated ruthenium OER catalyst. With this design, we successfully fabricated carbon‐coated nano‐size RuO_*x*_ particles (Si‐RuO_*x*_@C) with OER stability significantly improved up to 100 h in acidic solution, while the overpotential is also much lower than commercial RuO_2_. Interestingly this approach also led to Si doping into the lattice of RuO_*x*_ during calcination, as revealed by X‐ray absorption fine structure (EXAFS) and other analyses. Density functional theory (DFT) calculations showed that Si doping positively contributed to both reactivity and stability. Our approach provides a versatile way of generating various carbon‐coated nanoparticles for OER due to its modular nature.

## 2 Results and Discussion

**Figure 1:**
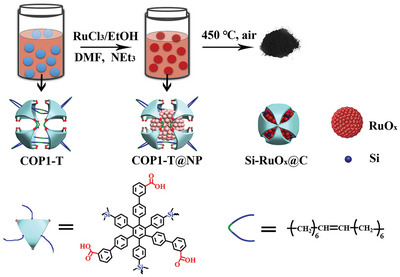
Preparation process of Si‐RuO_*x*_@C nanoparticles.

The synthetic route of caged trapped ruthenium oxide particle is shown in **Figure** **1**, and simply mixing of RuCl_3_ solution with COP1‐T solution in DMF led to an assembly COP1‐T and Ru ions. This process was monitored by the dynamic light scattering (DLS) analysis. As shown in Figure S2, Supporting Information, the particle size of Ru ion is slightly larger than that of COP1‐T, and the expansion of the cage is rationalized by the flexible nature of alkyl chains connecting the hexaphenyl subunits of COP1‐T. The cage expansion is consistent with our previous reports.^[^ ^37^ ^]^ After solvent removal, calcination at 450 °C under air for 6 h led to a black powder (Si‐RuO_*x*_@C).

**Figure 2:**
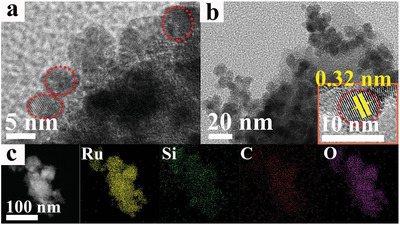
a,b) The HRTEM images of Si‐RuO_*x*_@C; the inset in b) shows the lattice of Si‐RuO_*x*_@C. c) STEM image and elemental mapping of Ru, Si, C, O.

The morphology of Si‐RuO_*x*_@C was characterized by high‐resolution transmission electron microscopy (HRTEM). As shown in **Figure** **2**a, RuCl_3_ was confined by cage precursors and followed by calcination which led to RuO_*x*_ nanoparticles in uniform size distribution with spherical shapes. In Figure 2b, the size of Si‐RuO_*x*_@C is ≈6 nm, which is reasonable considering the expansion of the cage upon encapsulation of Ru ions. Energy dispersive spectroscopy (EDS) mapping showed the existence of Si element in Si‐RuO_*x*_@C (Figure 2c), which was probably introduced through the C–Si bond scission during calcination. The local atomistic environment of Ru in Si‐RuO_*x*_@C was further probed with extended Fourier transforms of the X‐ray absorption fine structure (FT‐EXAFS) and X‐ray absorption near‐edge structure (XANES).^[^ ^39^ ^]^

**Figure 3:**
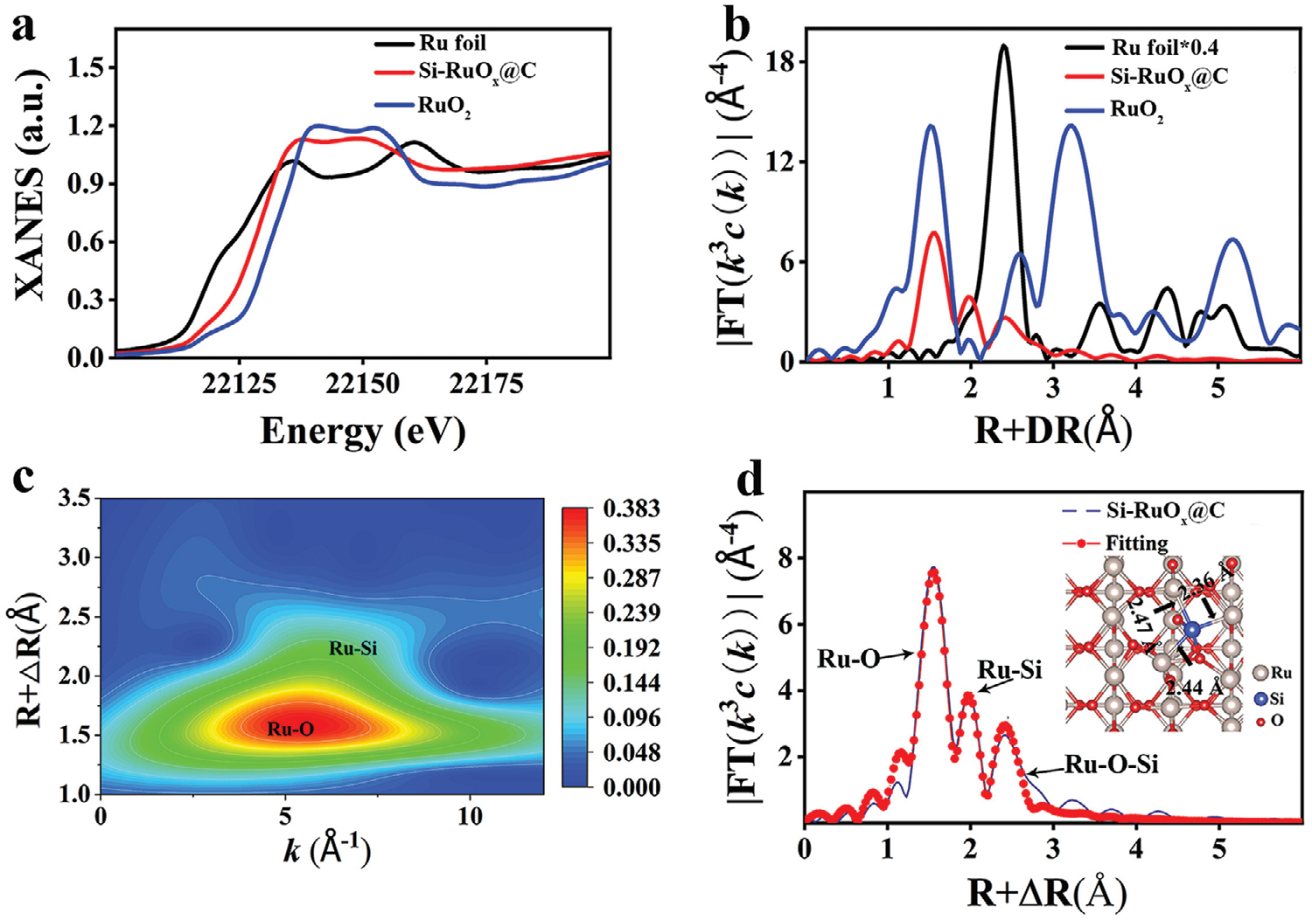
X‐ray absorption fine structures (XAFS) and X‐ray absorption near‐edge structure (XANES) spectra. a) Si‐RuO*_x_* @C L_3_‐edge XANES spectrum. b) FT‐EXAFS curves. c) WT‐EXAFS plots. d) FT‐EXAFS fitting curve of Si‐RuO*_x_* @C. Inset: schematic model.

The absorption edge of Si‐RuO_*x*_@C is located between those of Ru foil and RuO_2_ (**Figure** **3**a), and it indicates that the oxidation state of Ru is situated between Ru^0^ and Ru^4+^, and therefore Ru should be also bonded to elements with negativity much less than oxygen.^[^ ^40^ ^]^ In Figure 3b, the FT‐EXAFs analysis shows the main peak of Si‐RuO_*x*_@C at 2.01 Å, which is assigned to Ru–O bond.^[^ ^41^ ^]^ The peak at 2.35 Å is assigned to the bond length of Ru–Si bond, which is consistent with previous reports with the range between 2.30–2.52 Å.^[^ ^42^, ^43^, ^44^ ^]^ This bond is longer than the Ru–O (1.98 Å) bond in RuO_2_ and shorter than the Ru–Ru (2.68 Å) bond in metallic Ru. In addition, by comparing the wavelet transform (WT) results of Ru foil, there is no Ru–Ru characteristic peak in Si‐RuO_*x*_@C (Figure S3a,b,c,d), which can be found in Ru foil. These analyses are consistent with isoline results of WT‐EXAFS in which the main peaks correspond to Ru–O (4.3 Å^−1^) bond (Figure 3c and Figure S3e,f, Supporting Information). Based on these analyses, we, therefore, propose the structural model of Si doping into the RuO_2_ lattice (Ru‐intra‐Si), in which Ru is bonded to one Si and three O atoms (Figure 3d and Table S1, Supporting Information).

The cleavage of relatively weak Si–C bonds in the COP1‐T should be responsible for the formation Ru–Si bonds in Si–RuO*_x_* @C. It is known that Si‐C bond cleavage can be initialized by transition‐metal catalysts,^[^ ^45^, ^46^ ^]^ and this has particularly applications in organic reactions. In fact, María reported the Si–C bond cleavage by Ru clusters.^[^ ^47^ ^]^ We hypothesized that the COP1‐T trapped Ru atoms got involved in the Si‐C bond cleavage during the carbonization process, and this introduced the Ru–Si bond in Si‐RuO*_x_* @C.

**Figure 4:**
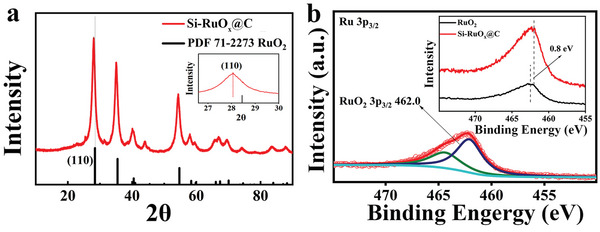
XRD and XPS spectra of Si‐RuO_*x*_@C. a) XRD patterns of Si‐RuO*_x_* @C. b) the XPS of Ru 3p regions.

As shown in **Figure** **4**a, powder X‐ray diffraction (XRD) shows that ruthenium oxide mainly exists in the form RuO_2_ without any special orientation, and this is consistent with the spherical shape of COP1‐T. Compared with the standard PDF#71‐2273 card, it is obvious that there are several lattice spacings of rutile RuO_2_ in Si‐RuO_*x*_@C, including mainly (110), (101), and some high‐index facets like (211) (Figure 4a). After Si intercalation, the (110) peak was shifted to a smaller angle (Figure 4a and Figure S4, Supporting Information), indicating the enlarged lattice which is similar to reported examples.^[^ ^48^, ^49^ ^]^ As shown in Figure S4a, Supporting Information, calcination is necessary for the formation of crystalline ruthenium oxide particle.

We also performed X‐ray photoelectron spectroscopy (XPS) to probe the oxidation state of Ru and Si atoms in Si‐RuO*_x_* @C. Since the peak of C 1s completely covers the area for Ru 3d_3/2_ and partially overlaps with Ru 3d_5/2_, it is inappropriate to quantitatively estimate the oxidation state of ruthenium from these regions, and therefore we focused on the binding energy for Ru 3p. As shown in Figure 4b, the Ru oxidation state of Si‐RuO*_x_* @C changed from Ru(III) to Ru(IV). Compared with the 3p_3/2_ peak 462.80 eV of commercial ruthenium oxide, the binding energy of ruthenium increases by 0.8 eV,^[^ ^50^ ^]^ which indicates that the introduction of Si leads to the change of the electronic structure of Ru (462.0 eV).^[^ ^43^, ^51^ ^]^ This XPS result matches the FT‐XANES results mentioned above.

**Figure 5:**
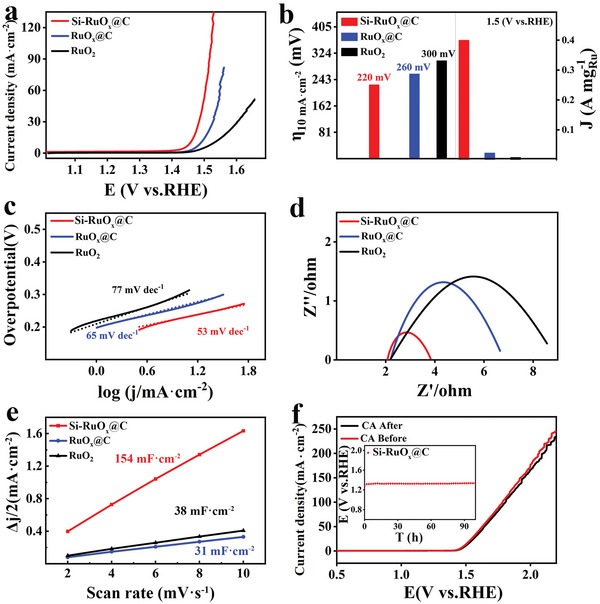
a) The OER polarization curves for Si‐RuO_*x*_@C, RuO_*x*_@C, and commercial RuO_2_ were acquired by linear sweep voltammetry (LSV) with a scan rate of 5 mV s^−1^ in 0.5 M H_2_SO_4_ at room temperature. b) Comparison between the mass activity of Si‐RuO*_x_* @C, RuO*_x_* @C and commercial RuO_2_ at 270 mV and the overpotentials required to achieve 10 mA cm^−2^ for Si‐RuO*_x_* @C, RuO*_x_* @C, and commercial RuO_2_. c) Tafel plots for Si‐RuO*_x_* @C, RuO*_x_* @C, and commercial RuO_2_. d) Electrochemical impedance spectroscopy (EIS) data for Si‐RuO*_x_* @C, RuO*_x_* @C, and commercial RuO_2_. Data were collected for the electrodes under OER overpotential of 10 mV. e) The electrochemical active surface area (ECSA) of Si‐RuO*_x_* @C, RuO*_x_* @C, and commercial RuO_2_. f) Comparison of LSV curves before (black) and after (red) chronoamperometry (CA) test. The insert is potential versus time (E–T) curves of Si‐RuO_*x*_@C recorded for 100 h at 10 mA cm^−2^.

The electrocatalytic OER performance of the catalyst was tested in 0.5 M H_2_SO_4_ electrolyte solution. Under the typical three‐electrode system, the OER activities of Si‐RuO*_x_* @C, RuO_*x*_@C, and commercial RuO_2_ (**Figure** **5**a) were compared. RuO*_x_* @C was prepared with carbon black as the support instead of COP1‐T (preparation details in supporting information). In the linear sweep voltammetry (LSV) curve, the minimum oxygen evolution overpotential of Si‐RuO*_x_* @C was 220 mV (10 mA cm^−2^), which was obviously more advantageous than 260 mV of RuO*_x_* @C and 300 mV of commercial RuO_2_. In addition, we also prepared RuO_*x*_ nanoparticles with the subunit molecules of the cage (L1 in the ref.[33]), and the only difference was the subunits are interconnected in COP1‐T through alkene bonds. As shown in Figure S7a, Supporting Information, the tying between different hexaphenylbenzene molecules significantly increased the activity. Interestingly, the Si‐RuO_*x*_@C also maintained OER activity in basic and nature conditions (Figure S5, Supporting Information). This result indicates that Si‐RuO*_x_* @C has better OER activity than both RuO*_x_* @C and RuO_2_. In addition, as shown in Figure 5b, when the working potential is 1.5 V (vs. RHE), Si‐RuO*_x_* @C provided a mass activity (MA) of 400.2 mA mg^−1^ Ru at an overpotential of 270 mV, which was 17.4 times and 47.6 times greater than RuO*_x_* @C and commercial RuO_2_, respectively. The content of Ru element in Si‐RuO_*x*_@C was ≈2.18%, estimated by ICP‐OES. As shown in Table S3, Supporting Information, Si‐RuO*_x_* @C is superior to most acidic OER catalysts.

In order to further prove the electrochemical OER performance of Si‐RuO*_x_* @C, the Tafel slope was used to judge on the dynamics of Si‐RuO*_x_* @C (Figure 5c). The Tafel slope of Si‐RuO_*x*_@C is only 53 mV dec^−1^, which is much lower than 65 mV dec^−1^ of RuO*_x_* @C and 77 mV dec^−1^ of RuO_2_, indicating that Si‐RuO*_x_* @C has a lower OER kinetic barrier. Electrochemical impedance spectroscopy (EIS) tests were carried out to investigate electrochemical reaction kinetics. As shown, the EIS of all catalysts displays a semicircle in the high‐frequency region and an inclined line in the low‐frequency region. The semicircle in the Nyquist plot represents the charge transfer resistance (*R*_ct_) of the electrode and electrolyte interface.^[^ ^52^ ^]^ It can be seen from Figure 5d that the *R*_ct_ of Si‐RuO_*x*_@C is the smallest, which indicates that Si‐RuO*_x_* @C has better electronic transmission capability compared with RuO*_x_* @C and RuO_2_.

We further evaluated the effect of calcination temperature and the amount of Si sources. As shown in Figure S6, Supporting Information, the optimal calcination temperature is 450 °C. Too low or too high calcination temperatures lead to inferior OER performances. The formation of carbon coating necessitates heating at high temperatures, but apparently, the carbon coating probably decomposed when the temperatures exceeded 450 °C. We further discussed the OER performance of different Si loading, and proved that additional Si doping deteriorates the OER activity (Figure S7b, Supporting Information).

In order to further understand the excellent OER electrochemical performance of Si‐RuO*_x_* @C, the electrochemical active surface area (ECSA) was measured according to the double‐layer capacitance (C_dl_) by cyclic voltammetry (CV) to determine the mechanism for high OER activity of Si‐RuO_*x*_@C. Figure 5e shows that the C_dl_ value of Si‐RuO*_x_* @C is 154 mF cm^−2^, which is much greater than 38 mF cm^−2^ of RuO*_x_* @C and 31 mF cm^−2^ of RuO_2_ respectively, indicating that Si‐RuO*_x_* @C has more electrochemical active sites relative to RuO*_x_* @C and RuO_2_ due to nanostructure.

Si‐RuO*_x_* @C exhibited remarkable stability during acidic OER. As shown in Figure S8, Supporting Information, after the stability test of 27 000 CV cycles, the overpotential at 10 mA cm^−2^ only increases by 10 mV. After OER at 10 mA cm^−2^ for 100 h, the overpotential still remained at ≈220 mV (Figure 5f). In fact, we measured the Ru content in the solution after 100‐hour stability test by ICP‐OES, and it showed <2% Ru was lost. In addition, we performed various analyses including HRTEM, SEM, EDS, and XPS of Si‐RuO*_x_* @C after CV stability test, and it was shown that Si‐RuO*_x_* @C remained almost intact in the challenging acidic environment (Figures S9, S10, S11, S12, Supporting Information). Given its great acidic stability, we also evaluate the hydrogen evolution reaction (HER) performance of Si‐RuO_*x*_@C in 0.5 M H_2_SO_4_, which has an overpotential of 200 mV. The overall water splitting (OWS) is also tested in acidic electrolyte solution. By comparing the performance of Si‐RuO*_x_* @C||Si‐RuO*_x_* @C and commercial Pt/C||RuO_2_, it can be seen from LSV that the cell voltage of Si‐RuO*_x_* @C||Si‐RuO_*x*_@C (1.66 V) is significantly less than the value of Pt/C||RuO_2_ (1.73 V) at 10 mA cm^−2^. These results indicate that Si‐RuO*_x_* @C has great potential in the development of full battery (Figure S13, Supporting Information).

We further performed DFT calculations to understand the underlying reason for the high activities of Si‐RuO_x_@C toward acidic OER (calculation details in the Experimental Section of Supporting Information). Previous studies have shown that there are two kinds of Ru sites on RuO_2_ (110), namely, coordination unsaturated sites, bridge sites,^[^ ^53^, ^54^ ^]^ and therefore the structural models of Ru‐O_4_‐Si and Ru‐O_5_‐Si were constructed by replacing Ru with Si at the coordination unsaturated sites and bridge sites on the RuO_2_ (110) (Figure S14, Supporting Information). The structure of Ru‐intra‐Si with direct Si coordination to Ru (Figure S14, Supporting Information) was proposed based on above analyses, especially considering the existence of unique Si‐Ru bonds.^[^ ^43^ ^]^ The comparison between Ru‐intra‐Si and Ru‐O_4_/O_5_‐Si would shed light on the origin of the superior performance of Si‐RuO*_x_* @C.

**Figure 6:**
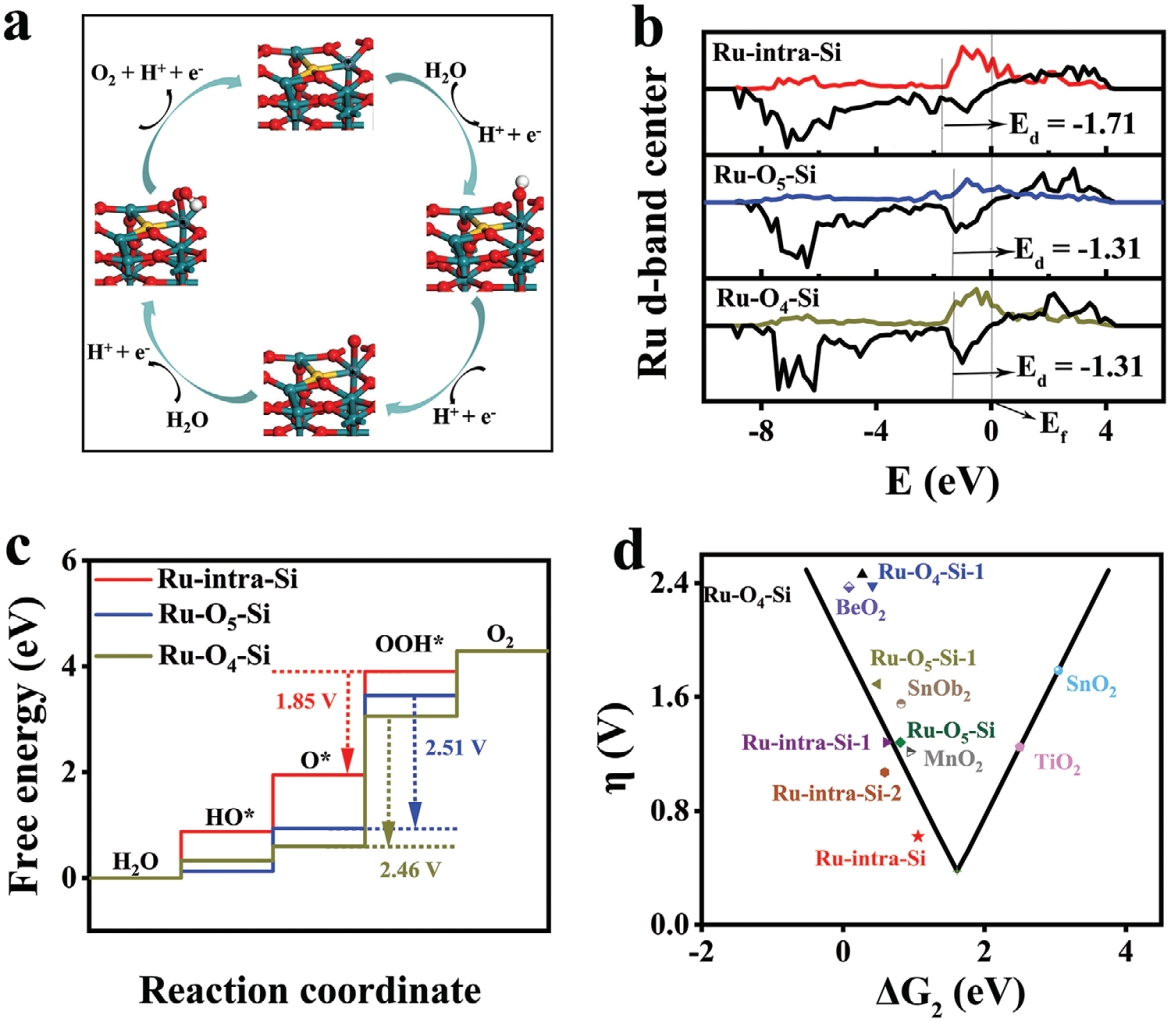
a) The four‐electron mechanism of Ru‐intra‐Si toward acidic OER. b) The d‐band center of Ru for Ru‐intra‐Si, Ru‐O_4_‐Si, and Ru‐O_5_‐Si. c) The Gibbs free energy diagram for Ru‐intra‐Si, Ru‐O_5_‐Si, and Ru‐O_4_‐Si. d) The calculated *η* for Si‐RuO_*x*_@C in the “volcano” plot of overpotential versus Δ*G*_2_ (Δ*G*_2_ = Δ*G*_OH*_ − Δ*G*_O*_)

There are three possible catalytic centers in Ru‐intra‐Si, and two centers in each of Ru‐O_4_‐Si and Ru‐O_5_‐Si (Figure S14, Supporting Information). All centers are evaluated with DFT calculations in terms of the free energies of various intermediates. **Figure** **6**a,c shows the free energies of various intermediates at the most feasible Ru active sites of Ru‐intra‐Si, Ru‐O_4_‐Si and Ru‐O_5_‐Si (model structure details in Figures S14, S15a, S16c, and S17e, Supporting Information). The catalytic OER performance is determined by the thermodynamic nature of the rate determining step, namely the transformation from O* to OOH*.^[^ ^18^, ^43^, ^55^, ^56^ ^]^ As shown in Figure 6c, all reaction steps of Ru‐intra‐Si, Ru‐O_4_‐Si and Ru‐O_5_‐Si are endothermic at zero potential. In the Ru‐intra‐Si, Ru‐O_4_‐Si, and Ru‐O_5_‐Si models, when the equilibrium potential is 1.23 V, some reaction processes are endothermic (Figures S15b, S16d, and S17f, Supporting Information). This is consistent with the fact that overpotentials are needed for OER of these catalysts. When the potential increased to 2.51 V and 2.46 V, the free energies for all intermediates of Ru‐O_4_‐Si and Ru‐O_5_‐Si models run downhill respectively, while only 1.85 V is required to make all intermediates become downhill in Ru‐intra‐Si model. The decrease of required potentials demonstrate that Si doping could facilitate the oxygen evolution process (Figures S15b, S16d, and S17f, Supporting Information).

Electronic interaction between the bonding and antibonding orbitals of adsorbed oxygen species and the transition metals.^[^ ^57^ ^]^ Previous studies have demonstrated that the Ru‐O molecular orbitals around the Fermi level, *E*_f_, are only of the antibonding states,^[^ ^58^ ^]^ and therefore the d‐band center (*E*_d_) can used to estimate the position of antibonding orbitals.^[^ ^53^ ^]^ By the projected density of states (PDOS) of d‐band in Ru, the E_d_ values of Ru‐O_4_‐Si, Ru‐O_5_‐Si, and Ru‐intra‐Si are −1.31, −1.31 and −1.71 eV respectively relative to E_f_ (Figure 6b), which unambiguously shows that the Si insertion makes the E_d_ more negative due to interaction between p orbital of Si and d orbital of Ru (Figures S18, S19, and Figure S20).^[^ ^59^ ^]^ Ru‐intra‐Si exhibits higher occupied states than Ru‐O_4_‐Si and Ru‐O_5_‐Si near the *E*_f_, corresponding to the promoted electron transport, subsequently leading to enhanced conductivity^[^ ^57^ ^]^; which was supported by EIS analysis (Figure 5d). The lowering of E_d_value lowered the antibonding states for absorbed species, and therefore weakened the corresponding Ru‐bond, which is known to be beneficial for OER efficiency.^[^ ^58^ ^]^ In addition, as illustrated in Figure 6d, the plot of the overpotential *η* of OER versus the standard free energy ΔG_2_ exhibits a volcano shape (the values of MnO_2_, SnOb_2_, BeO_2_, TiO_2,_ and SnO_2_ were taken from ref.[59]), and the position of best Ru‐intra‐Si sites was close to the optimal turning point, and therefore confirming the origin of the high reactivity of Ru‐intra‐Si.

Furthermore, the differential charge density with Ru as the catalytic center can explain the oxidation and corrosion resistance of Si‐RuO_*x*_@C.^[^ ^34^ ^]^ It is proved that RuO_*x*_ can obtain an appropriate amount of electrons from Si (Figure S21, Supporting Information). This result was further confirmed by Bader charge. The average charge density of Ru around Si is 1.179 electrons (|e|), which is significantly lower than that of Ru^4+^ 1.726 electrons (|e|) in RuO_2_ by 0.547 electrons (|e|). This further proves that Si can act as an electron library to provide Ru electrons, thereby enhancing the oxidation resistance and corrosion resistance of RuO*_x_*.^[^ ^43^ ^]^ Therefore, the insertion of Si into the (110) side of RuO_2_ can not only effectively improve the OER activity, also make an important contribution to the stability of Si‐RuO*_x_* @C.

## 3 Conclusion

In conclusion, the Si‐RuO*_x_* @C from nano organic cage can not only have high OER activity, but also have excellent stability, especially in acidic conditions. When the working current density is 10 mA·cm^−2^, the oxygen evolution overpotential of Si‐RuO*_x_* @C is 80 mV lower than that of commercial RuO_2_ (300 mV). Si‐RuO*_x_* @C can maintain constant oxygen evolution for 100 hours in acid electrolyte, and the CV energy is still stable after running 27000 cycles. The DFT calculations show that the Si insertion makes the d‐band center more negative, and optimizes the Gibbs free energy of adsorbed oxygen state in the rate‐determining step. In addition, the presence of Si around Ru as the electron reservoir increase the oxidation resistance of Ru centers for high stability. These findings pave an efficient way for rational design of electrocatalysts with high catalytic activity and stability operated under harsh conditions.

## Conflict of Interest

The authors declare no conflict of interest.

## Supporting information

Supporting Information

## Data Availability

The data that support the findings of this study are available from the corresponding author upon reasonable request.
