# Supplementary material for: Nano Si‐Doped Ruthenium Oxide Particles from Caged Precursors for High‐Performance Acidic Oxygen Evolution
Source: Adv Sci (Weinh). 2023 Feb 21;10(13):2207429. doi: 10.1002/advs.202207429 (PMC10161032; doi:10.1002/advs.202207429)
Supplement: Supplementary file 1 — Supporting Information [file ADVS-10-2207429-s001.pdf]

Supporting Information

Nano Si-doped Ruthenium Oxide Particles from Caged Precursors for High-Performance Acidic Oxygen Evolution

Chunxiang Liu[a], Yunbo Jiang[b], Teng Wang[a,c], Qiaosheng Li[a], and Yuzhou Liu[a,c,d]**

a School of Chemistry, Beihang University, Beijing, 100191, China
b State Key Laboratory for Advanced Technologies for Comprehensive Utilization of Platinum Metals Sino-Platinum Metals Co. Ltd., Kunming 650221, China
c Beijing Advanced Innovation Center for Biomedical Engineering, School of Chemistry, Beihang University, Beijing, 100191, China
d Research Department, Shenyunzhihe Co. Ltd., Qidian Center, FL 10, Energy East Rd #1, Changping District, Beijing, 102206, China

E-mail: tengwang@buaa.edu.cn; liuyuzhou@buaa.edu.cn;

Table of Contents

S1 Materials and Methods S3

S1-1: Materials and Equipments S3

S1-2: Materials Preparation S3

Preparation of RuO_x/C S4

Preparation of Si-RuO_x@C S4

Preparation of RuO_x/L1 .... S5

Preparation of Si_3-RuO_x@C S5

Preparation of Si_4-RuO_x@C S6

S1-3: Material Characterization S6

Electrochemical experiments S6

DFT calculations S7

S2 Figures and Tables S8

S3 Modeling S19

S3-1: Optimized structure information of Ru-intra-Si S19

S3-2: Optimized structure information of Ru-O_5-Si S20

S3-3: Optimized structure information of Ru-O_4-Si S22

REFERENCES S24

S1 Materials and Methods

S1-1: Materials and Equipments

All materials were used as received without further purification, unless otherwise noted. Ethanol was dried with Mg turnings.

All manipulations were carried out in air atmosphere, unless otherwise noted. The high resolution transmission electron microscopy (HRTEM) experiments were recorded on a Hitachi 7650 electron microscope. The scanning electron microscopy (SEM) and energy dispersive X-ray spectrometry (EDS) were recorded on a Jeol JSM-7500F field emission scanning electron microscope. The IR spectra were recorded with KBr pellets on a Bruker EQUINOX 55 FT-spectrometer in the range of 4000−500 cm^{-1}. Powder X-ray diffraction (PXRD) patterns were recorded on Bruker D8 Advance analytical diffractometers for Cu K = radiation (λ = 1.5406 Å). ICP-OES measurement was performed using Optima-7000 DV spectrometer. Raman spectroscopy was conducted on a Jobin Yvon LabRAM HR 800 instrument with a 532 nm excitation laser at a power of around 0.8 mW.

Data reduction, data analysis, and EXAFS fitting were performed with the Athena and Artemis software packages. The energy calibration of the sample was conducted through a standard Ru foil, which was simultaneously measured as a reference. For EXAFS modeling, EXAFS of the Ru foil is fitted, and the obtained amplitude reduction factor S_0^2 value (0.890) was set in the EXAFS analysis to determine the coordination numbers (CNs) in the Ru-O/Si scattering path. For Wavelet Transform analysis, the $\chi(k)$ exported from Athena was imported into the Hama Fortran code. The parameters were listed as follow: R range, 1 – 3.5 Å, k range, 0 – 13.0 $Å^{-1}$ for sample (0 – 13.0 $Å^{-1}$ for Ru foil and RuO_2); k weight, 2; and Morlet function with κ = 10, σ = 1 was used as the mother wavelet to provide the overall distribution.

S1-2: Materials Preparation

Metal sources $RuCl_3 \cdot xH_2O$ and sodium alginate were purchased from Aladdin. Nafion® perfluorinated resin solution containing 5% Nafion® was purchased from Sigma-Aldrich. Ultrapure water with resistivity > 18 MΩ·cm^{-1} was used.

Preparation of RuO_x/C

The $RuCl_3$ (56 mg) was dissolved in absolute ethanol (8 mL) and stirred for 30 min to give a transparent red solution. To the ultra-dry DMF (72 mL) was added commercial acetylene black (96 mg), and the ultrasonic dispersing of this suspension was performed for 30 min. A 20 mL vial was charged with the above-mentioned $RuCl_3$ solution (1 mL) and acetylene black dispersion (12 mL). The vial was sealed under air and placed in drying oven at 85 °C for 72 h. The mixture was then cooled to room temperature and dried under vacuum, and the residual black powder was transferred to the muffle furnace at 450 °C for 6 h. The RuO_x/C was obtained as a black powder.

Preparation of Si-RuO_x@C

The $RuCl_3$ (56 mg) was dissolved in absolute ethanol (8 mL) and stirred for 30 min to give a transparent red solution. As an organic cage for which we have reported the synthetic method,[1] COP1-T (96 mg) was dissolved in ultra-dry DMF (12 mL) and stirred at 150 mL round bottom flask for 30 min. A 20 mL vial was charged with the above-mentioned $RuCl_3$ solution (1 mL), COP1-T solution (12 mL), and triethylamine (12 μL) (the Si/Ru mass ratio is 1 : 8.5). The vial was sealed under air and placed in drying oven at 85 °C for 72 h. Then the reaction flask was cooled to room temperature, dried, and then transferred to a muffle furnace. In order to explore the influence of temperature on OER performance, we adopted the same method to prepare catalysts at different temperatures at 150 °C, 250 °C, 350 °C, 450 °C and 550 °C respectively.

Preparation of RuO_x/L1

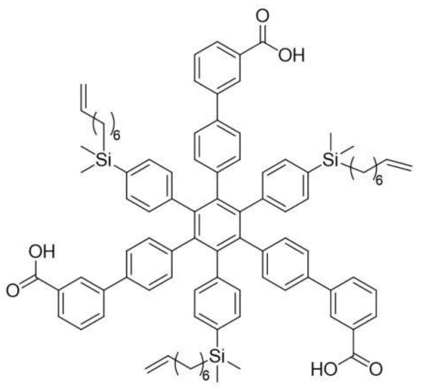

L1

Figure S1.The structure of L1.

The hexaphenylbenzene carboxylic acid molecule (L1) was prepared according to our previous report. The $RuCl_3$ (56 mg) was dissolved in absolute ethanol (8 mL) and stirred for 30 min to give a transparent red solution. To an ultra-dry DMF solution (72 mL) of (96mg) L1[1] and the solution was stirred for 30 min. A 20 mL vial was charged with the above-mentioned $RuCl_3$ solution (1 mL), the COP1-T solution with silane (12 mL), and triethylamine (12 μL). The vial was sealed under air and placed in drying oven at 85 ℃ for 72 h. Then the reaction flask was cooled to room temperature, dried, and then transferred to a muffle furnace at 450 °C for 6 h to give RuO_x@L1 as a black powder.

Preparation of Si_3-RuO_x@C

The $RuCl_3$ (56 mg) was dissolved in absolute ethanol (8 mL) and stirred for 30 min to give a transparent red solution. To an ultra-dry DMF solution (72 mL) of COP1-T (96 mg) was added chloro(dimethylphenyl)silane (6.58 mg) and the solution was stirred for 30 min. A 20 mL vial was charged with the above-mentioned $RuCl_3$ solution (1 mL), the COP1-T solution with silane (12 mL), and triethylamine (12 μL) (the Si/Ru mass ratio is 2 : 8.5). The vial was sealed under air and placed in drying oven at 85 °C for 72 h. Then the reaction flask was cooled to room temperature, dried, and then transferred to a muffle furnace at 450 °C for 6 h to give Si_3-RuO_x@C as a black powder.

Preparation of Si_4-RuO_x@C

The $RuCl_3$ (56 mg) was dissolved in absolute ethanol (8 mL) and stirred for 30 min to give a transparent red solution. To an ultra-dry DMF solution (72 mL) of COP1-T (96 mg) was added chloro(dimethylphenyl)silane (13.16 mg) and stirred for 30 min. A 20 mL vial was charged with the above-mentioned $RuCl_3$ solution (1 mL), the COP1-T solution with silane (12 mL), and triethylamine (12 μL) (the Si/Ru mass ratio is 3 : 8.5). The vial was sealed under air and placed in drying oven at 85 °C for 72 h. Then the reaction flask was cooled to room temperature, dried, and then transferred to a muffle furnace at 450 °C for 6 h to give Si_4-RuO_x@C as a black powder.

S1-3: Material Characterization

Electrochemical experiments

Cyclic voltammetry (CV) measurements were carried out in a standard one-compartment cell equipped with a carbon rob counter electrode [Ag/AgCl (3 M KCl)], a reference electrode (Hg/HgO), and a glassy carbon (GC) working electrode. The measurements were performed in deionized water with 0.5 M H_2SO_4, 1 M KOH and 1 M phosphate buffered saline (PBS) as the supporting electrolyte. The data is collected and processed by the Shanghai electrochemical analyzer (CHI760e) at 25 °C. The linear sweep voltammetry (LSV) for OER were recorded in 0.5 M H_2SO_4 at the potential range from 1.0 − 1.6 V (vs.RHE). The LSVs for HER were recorded in 0.5 M H_2SO_4 at the potential range of -2 − 0 V (vs. RHE). The electrochemical surface area was determined by a double layer capacitance (C_{dl}) method, in which the double-layer charging currents were measured in 0.5 M H_2SO_4 at the potential range from 0.1 − 0.2 V and at the scanning rates range from 10 to 100 $mV \cdot s^{-1}$. Cyclic voltammetry curves were measured at different scan rates (20 $mV \cdot s^{-1}$, 40 $mV \cdot s^{-1}$, 60 $mV \cdot s^{-1}$, 80 $mV \cdot s^{-1}$ 100 $mV \cdot s^{-1}$). In the overall water splitting (OWS), two Si-RuO_x@C electrodes acted as the negative electrode for HER and the positive electrode for OER, respectively. The LSV was recorded at a scan rate of 5 $mV \cdot s^{-1}$. For the benchmark control, commercial Pt/C was used as the negative electrode and commercial RuO_2 as the positive electrode.

For the preparation of working electrode, Si-RuO_x@C catalyst (5 mg) was dispersed in the mixture of Nafion® (50 μL, 5 wt.%) solution and ethanol (450 μL). Then a homogenous catalyst ink was obtained by ultrasonic dispersion. The resulting ink (31 μL) was pipetted onto a glassy carbon disk (0.196 cm^2). The mass loading of catalyst is 1.6 $mg \cdot cm^{-2}$. For comparison, working electrodes of commercial RuO_2 and RuO_x@C with mass loading of 1.6 $mg \cdot cm^{-2}$ were also prepared by the same procedure.

For the durability test of the catalyst supported on carbon fiber, the Si-RuO_x@C catalyst (3 mg) was dispersed in 0.5 mL mixture of ethanol (0.25 mL) and water (0.25 mL) with Nafion® solution (10 μL, 5 wt.%, DuPont) to form homogeneous ink by ultrasonic dispersion. Then the ink was added dropwise onto the surface of carbon paper with 1 cm^2 by using a micropipette and dried at room temperature. The final loading for all catalysts is 1.6 $mg \cdot cm^{-2}$ on carbon paper.

All potentials were referenced to the reversible hydrogen electrode (RHE) by the equations as follows (Eq.1 and Eq. 2):

$$\mathrm{E(RHE)} = \mathrm{E(Ag/AgCl, 3\ M\ KCl - filled)} + 0.059\, pH + 0.197 \qquad \text{(Eq. 1)}$$

$$\mathrm{E(RHE)} = \mathrm{E(Hg/HgO, 1\ M\ KOH - filled)} + 0.059\, pH + 0.098 \qquad \text{(Eq. 2)}$$

All electrochemical data were not subjected to IR compensation.

DFT calculations

Vienna Ab-initio Simulation Package (VASP) was used for all calculations with Revised Perdew-Burke-Ernzerhof (RPBE) functional for the exchange-correlation term and the projector augmented wave method[2-9].

The cutoff energies for all calculations were set to 520 eV. To consider the impact of van der Waals interactions between graphene and RuO_2 surface, the dispersion interaction corrected DFT (DFT-D_2) method was introduced in the structure optimization.[10] All structures were fully relaxed to the ground state and spin-polarization was considered in all calculations. The convergences of energy and force were set to 1×10^{-5} eV and 0.01 $eV \cdot Å^{-1}$, respectively. The vacuum space value is set to 20 Å to separate the interaction between neighboring slabs. As for OER, the free energies of the intermediates at 298.15 K were obtained by the following

equation (Eq. 3):

$$\Delta G = \Delta E + \Delta ZPE - T\Delta S + eU \quad \text{(Eq. 3)}$$

In this equation, ΔE is the binding energy of adsorption species HO*, O* and HOO*, ΔZPE, ΔS and U are the zeropoint energy changes, entropy changes and applied potentials, respectively. Furthermore, the energy barrier (η) was calculated by the following equation (Eq. 4):

$$\eta = (\Delta G_{OOH*} - \Delta G_{O*} - 1.23)/e \quad \text{(Eq. 4)}$$

In this equation, the asterisk represents active site, and 1.23 V stands for the equilibrium potential under the standard condition.

For pure RuO_2 catalyst, the RuO_2 (110) surface with 4 × 5 supercell was chosen as the structure. For Si introduced RuO_2 (110), one of the surface Ru was replaced by Si atom. In order to minimize the influence of lattice mismatch, we have carefully chosen the supercells for RuO_2 and the models are listed in S3-1, S3-2, S3-3.

S2 Figures and Tables

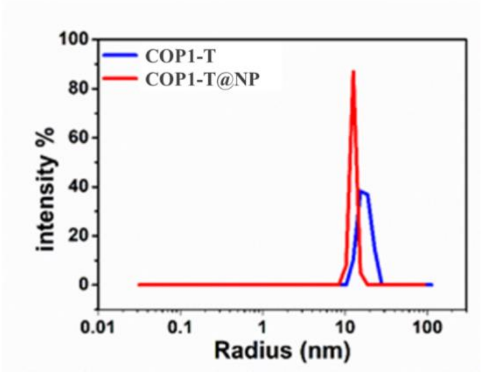

Figure S2. The DLS of COP1-T and COP1-T@NP.

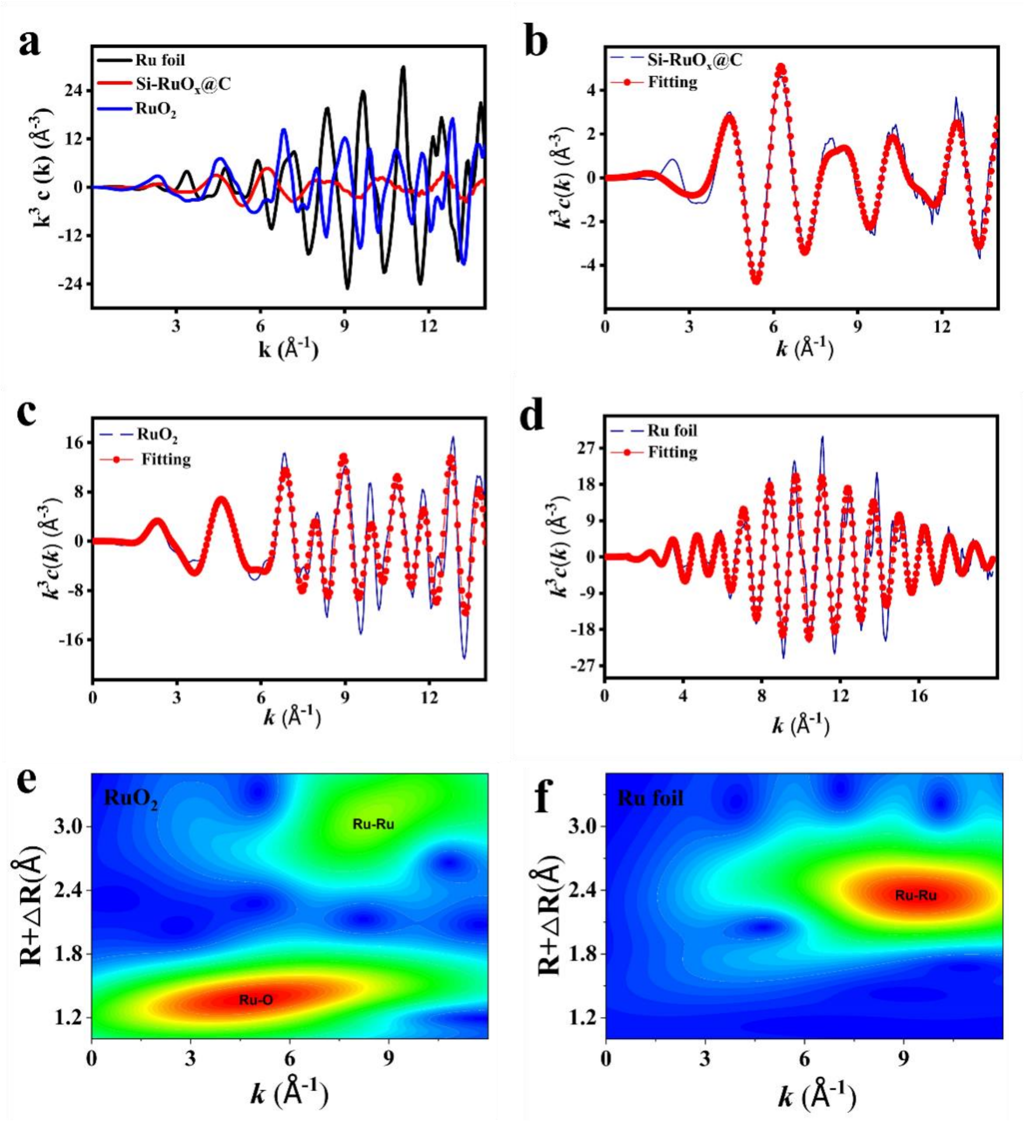

Figure S3. a) K-edge EXAFS spectrum of K-space Si-RuO_x@C, Ru foil and RuO_2. b) K-edge EXAFS fitting curve of K-space Si-RuO_x@C. c) EXAFS fitting curve of RuO_2. d) EXAFS fitting curve of Ru foil. e) and f) WT-EXAFS spectrum of Ru foil and RuO_2, respectively.

Table S1. EXAFS fitting parameters at the Ru K-edge for various samples（$S_0^2 = 0.790$）

Sample	Shell	CN^a	R (Å)^b	σ^2 (Å^2)^c	ΔE_0 (eV)^d	R factor
Ru foil	Ru-Ru	12*	2.68 ± 0.01	0.0044 ± 0.0003	5.7 ± 1.0	0.0046
RuO_2	Ru-O	5.8 ± 0.6	1.98 ± 0.01	0.0033 ± 0.0007	-0.2 ± 1.2	0.0057
	Ru-Ru	3.5 ± 0.7	3.12 ± 0.01	0.0047 ± 0.0009		
	Ru-Ru	10.1 ± 2.0	3.56 ± 0.01	0.0047 ± 0.0009		
Ru	Ru-O	2.7 ± 0.2	2.01 ± 0.01	0.0007 ± 0.0004	-13.5 ± 0.8	0.0104
	Ru-Si	1.4 ± 0.3	2.35 ± 0.01	0.0022 ± 0.0009		
	Ru-O-Si	3.8 ±1.2	2.84 ± 0.01	0.0067 ± 0.0022		

^{a}*CN*, coordination number; ^{b}*R*, distance between absorber and backscatter atoms; $^c\sigma^2$, Debye-Waller factor to account for both thermal and structural disorders; $^d\Delta E_0$, inner potential correction; *R* factor indicates the

goodness of the fit. S_0^2 was fixed to 0.790, according to the experimental EXAFS fit of M foil by fixing CN as the known crystallographic value. Fitting range: $3.0 \leq k$ (/Å) ≤ 17.8 and $2.0 \leq R$ (Å) ≤ 3.0 (Ru foil); $3.0 \leq k$ (/Å) ≤ 13.0 and $1.0 \leq R$ (Å) ≤ 3.8 (RuO_2); $3.0 \leq k$ (/Å) ≤ 14.0 and $1.0 \leq R$ (Å) ≤ 3.0 (Ru). A reasonable range of EXAFS fitting parameters: $0.700 < S_0^2 < 1.000$; $CN > 0$; $\sigma^2 > 0$ Å^2; $\Delta E_0 < 15$ eV; R factor < 0.02.

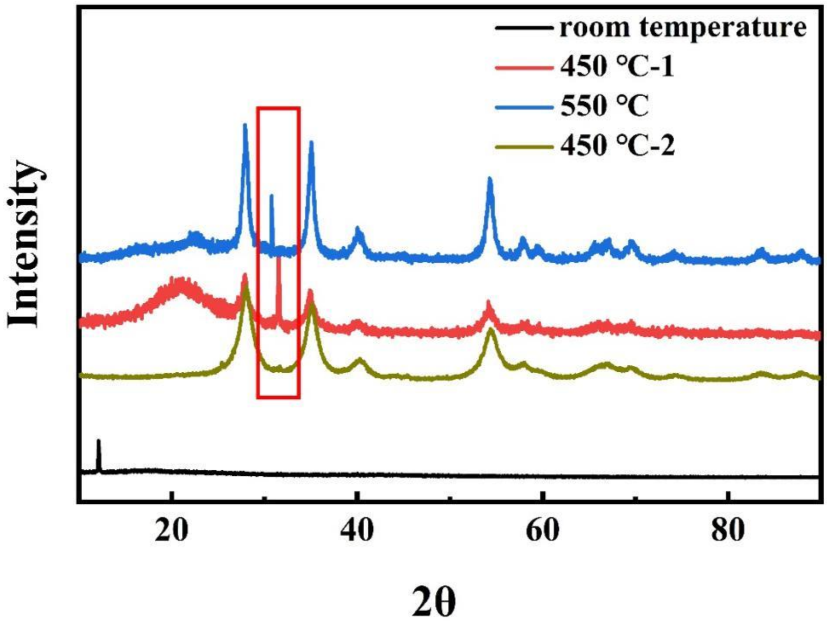

Figure S4. XRD diagram of Si-RuO_x@C calcined at different temperatures. Two tests were done for the samples of Si-RuO_x@C, indicating that the peaks around 30° (in the red box) are attributed to unindentified impurities introduced during the sample preparation other than from the catalysts. 450 °C-1 represents the first test, whereas 450 °C-2 represents the second test.

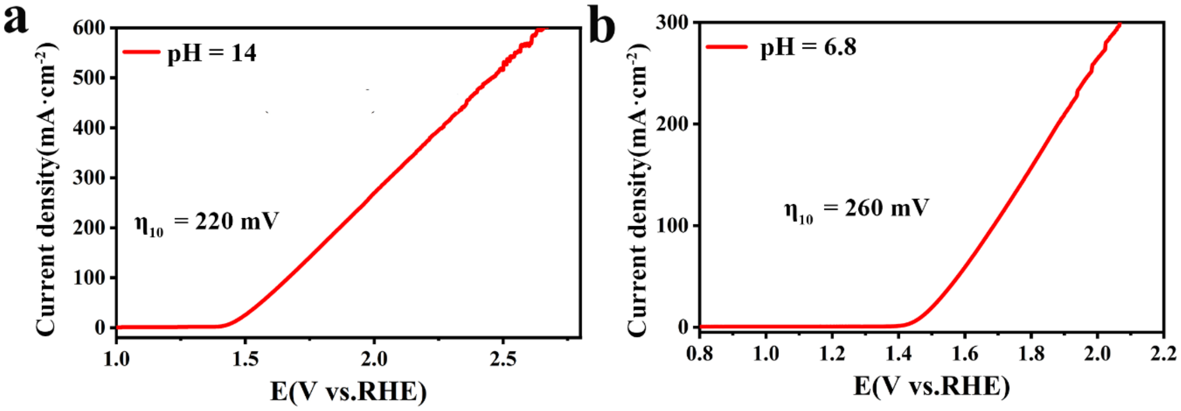

Figure S5. a) LSV of Si-RuO_x@C in 1 M KOH (pH = 14). b) LSV of Si-RuO_x@C in 1 M PBS (pH = 6.8).

Table S2. Comparison of OER performance for Si-RuO_x@C with some benchmark Ir-based oxides in acidic media.

Catalyst	Electrolyte solution	Overpotential (mV)	Stability	Loading amount ($mg \cdot cm^{-2}$)	Ref.
Si-RuO_x@C	0.5 M H_2SO_4	$\eta_{10} = 220$	100 h (10 $mA \cdot cm^{-2}$)	0.017 (Ru)	This work
RuO_x@C	0.5 M H_2SO_4	$\eta_{10} = 260$	–	1.6	This work
RuO_2	0.5 M H_2SO_4	$\eta_{10} = 300$	–	1.6	This work
6H-$SrIrO_3$	0.5 M H_2SO_4	$\eta_{10} = 248$	30 h (10 $mA \cdot cm^{-2}$)	0.90	[11]
IrO_2-NPs	0.5 M H_2SO_4	$\eta_{10} = 297$	4.0 h (20 $mA \cdot cm^{-2}$)	0.20 (IrO_2)	[12]
Ru@IrO_x	0.05 M H_2SO_4	$\eta_{10} = 282$	2 h (10 $mA \cdot cm^{-2}$)	0.05	[13]
IrNi-NCs	0.1 M $HClO_4$	–	2 h (5 $mA \cdot cm^{-2}$)	0.0125 (Ir)	[14]
Li-IrO_x	0.5 M H_2SO_4	$\eta_{10} = 300$	10 h (10 $mA \cdot cm^{-2}$)	0.05	[15]
$Ba_4PrIr_3O_{12}$	0.1 M $HClO_4$	$\eta_{10} = 278$	10 h (10 $mA \cdot cm^{-2}$)	0.56	[16]
IrO_2-RuO_2@Ru	0.5 M H_2SO_4	$\eta_{10} = 281$	2 h (1.6 V)	0.100	[17]
Co-IrCuONC	0.1 M $HClO_4$	$\eta_{10} = 293$	–	0.020 (Ir)	[18]
$W_{0.57}Ir_{0.43}O_{3-\delta}$	1.0 M H_2SO_4	$\eta_{10} = 370$	0.55 h (10 $mA \cdot cm^{-2}$)	–	[19]
Ir-W-B alloy	0.5 M H_2SO_4	$\eta_{10} = 291$	800 h (100 $mA \cdot cm^{-2}$)	0.079 (Ir)	[20]
IrO_x/$SrIrO_3$	0.5 M H_2SO_4	$\eta_{10} = 290$	30.0 h (10 $mA \cdot cm^{-2}$)	–	[21]
Pt/IrO_2	0.5 M H_2SO_4	$\eta_{10} = 348$	–	0.38	[22]
Ir_3Cu	0.1 M $HClO_4$	$\eta_{10} = 298$	12.0 h (5 $mA \cdot cm^{-2}$)	–	[23]
Pd-Ir-Pd	0.1 M $HClO_4$	$\eta_{10} = 372$	–	0.017	[24]
IrO_x-Ni	0.5 M H_2SO_4	$\eta_{10} = 290$	100 h (2 $mA \cdot cm^{-2}$)	0.13 (Ir)	[25]

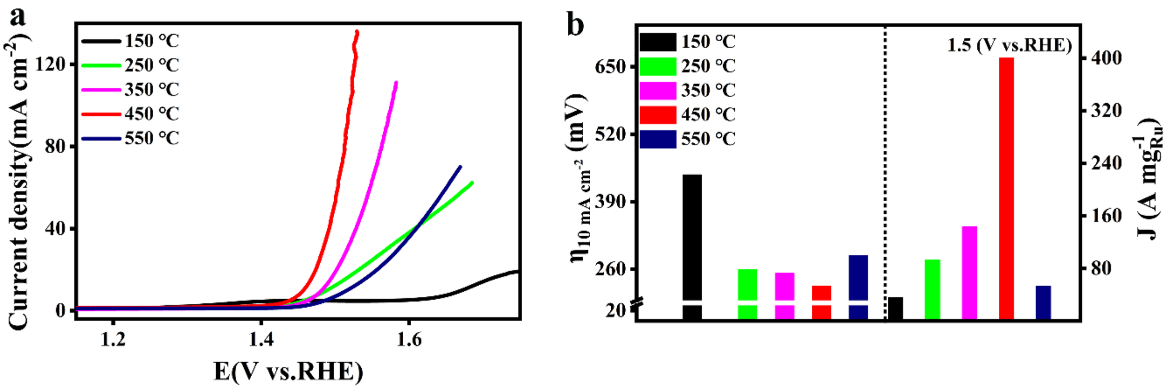

Figure S6. a) LSV test of Si-RuO_x@C at different temperatures in 0.5 M H_2SO_4. b) Mass activity and overpotential.

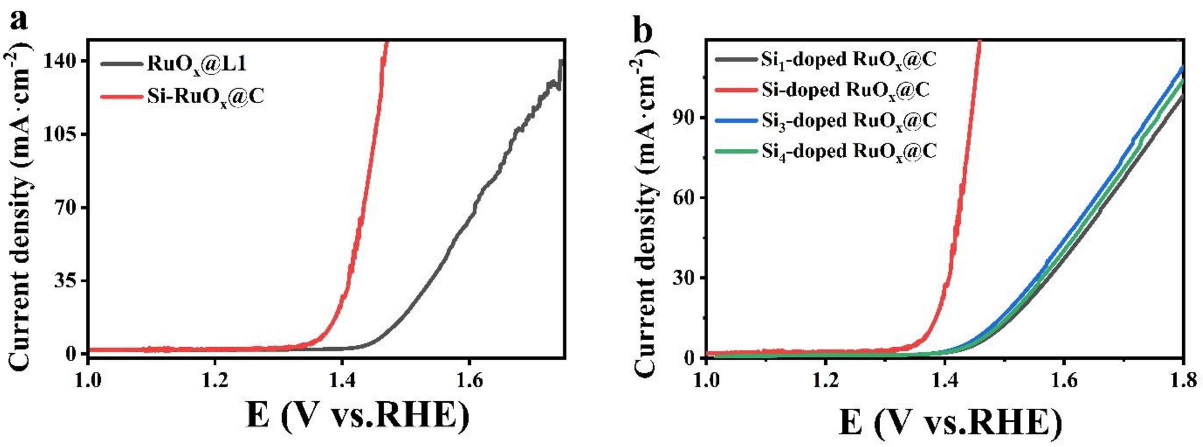

Figure S7. (a) The comparison between the OER performance of RuO_x@L1 and Si-RuO_x@C. (b)Si-RuO_x@C OER test with different Si contents. The RuO_x@C sample contains no Si. The Si/Ru mass ratio of Si-RuO_x@C is 1 : 8.5; the Si/Ru mass ratio of Si_3-RuO_x@C is 2 : 8.5; the Si/Ru mass ratio of Si_4-RuO_x@C is 3 : 8.5.

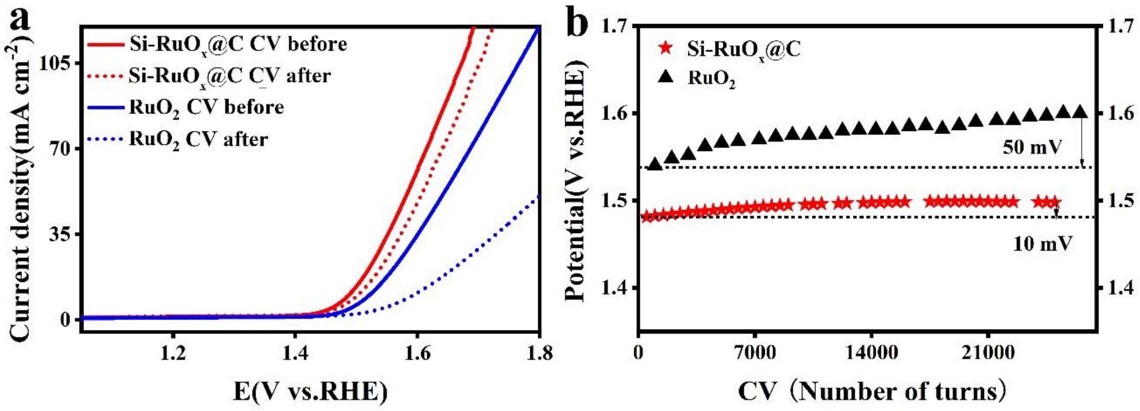

Figure S8. The Si-RuO_x@C of CV stability test. a) the LSV of before and after CV test. b) During the CV test, the potential changes with the number of scanning cycles at 10 mA·cm^{-2}.

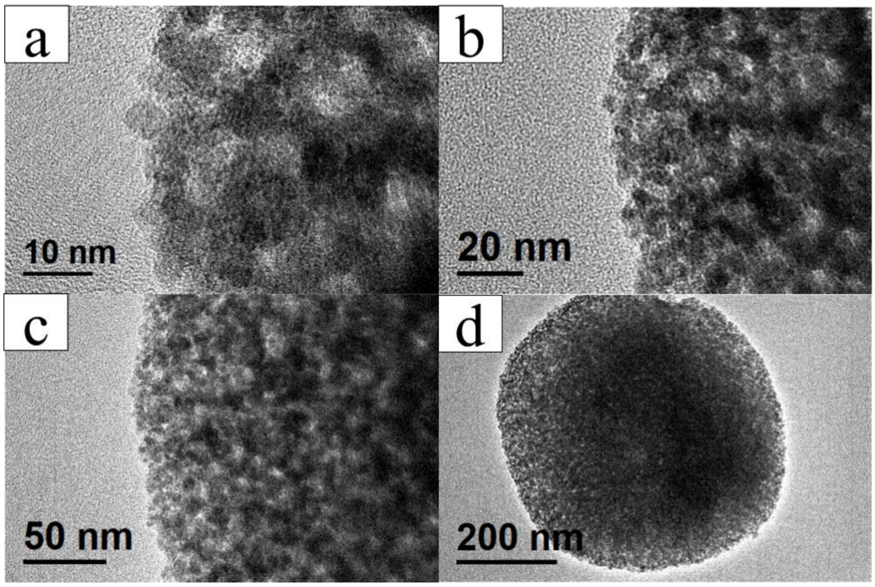

Figure S9. HRTEM diagrams of Si-RuO_x@C after CV test. a) to d) The data were collected on various scales.

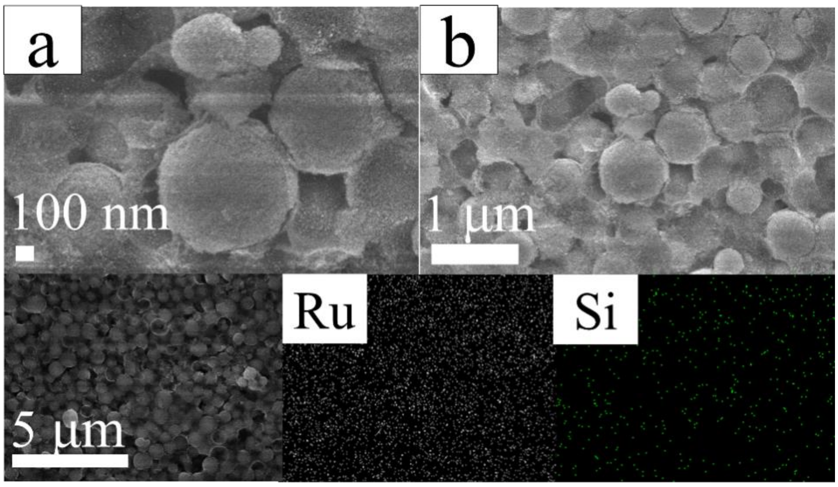

Figure S10. SEM and mapping diagrams of Si-RuO_x@C after CV test.

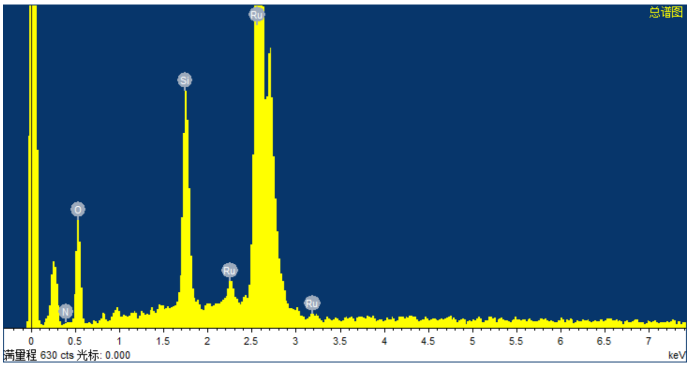

Figure S11. EDS diagram of Si-RuO_x@C after CV test.

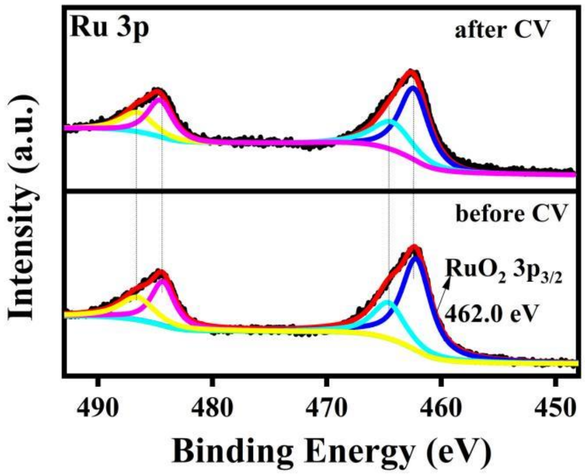

Figure S12. The XPS of Si-RuO_x@C comparison before and after CV stability test.

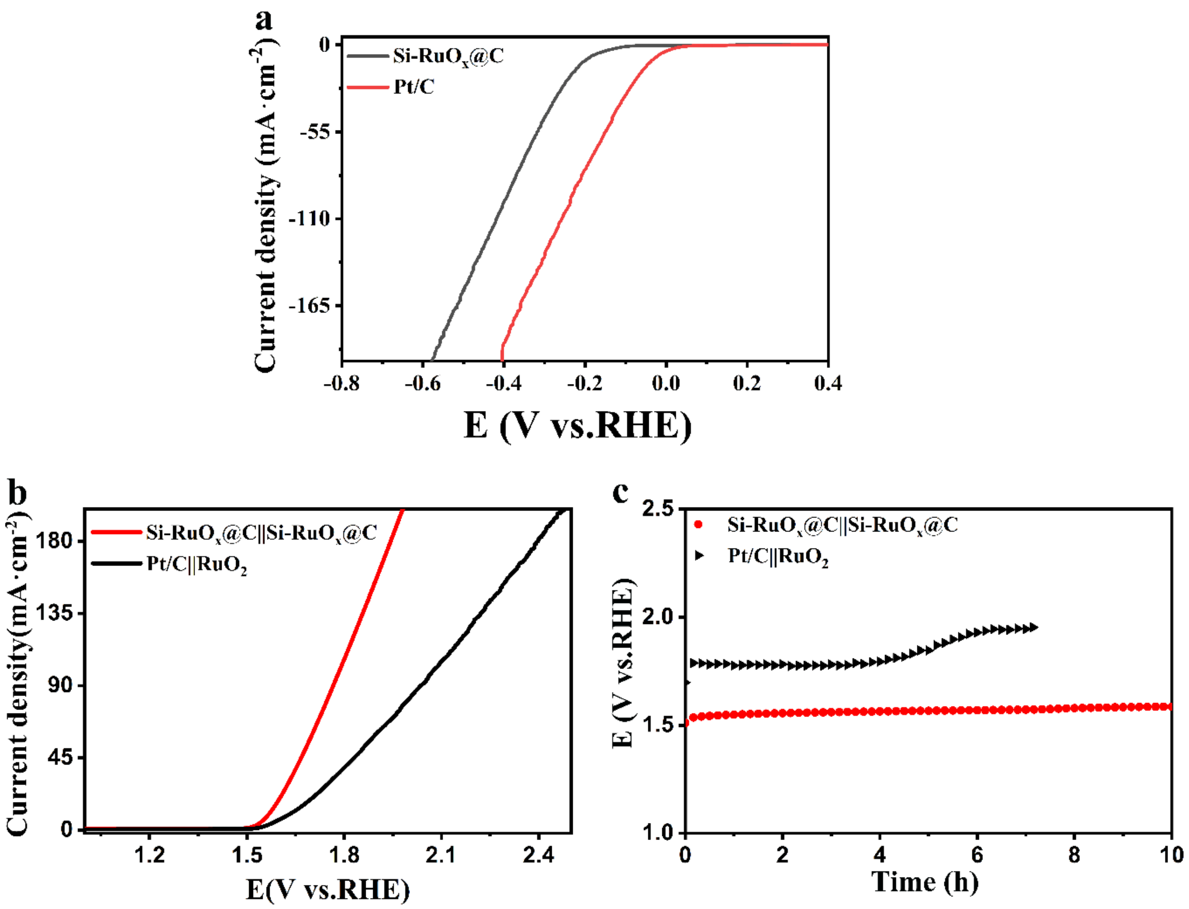

Figure S13. a) HER performance test of Si-RuO_x@C in 0.5 M H_2SO_4. b) OWS LSV curves without iR compensation. c) Stability test of Si-RuO_x@C||Si-RuO_x@C and Pt/C||RuO_2 for OWS at j = 10 mA·cm^{-2}.

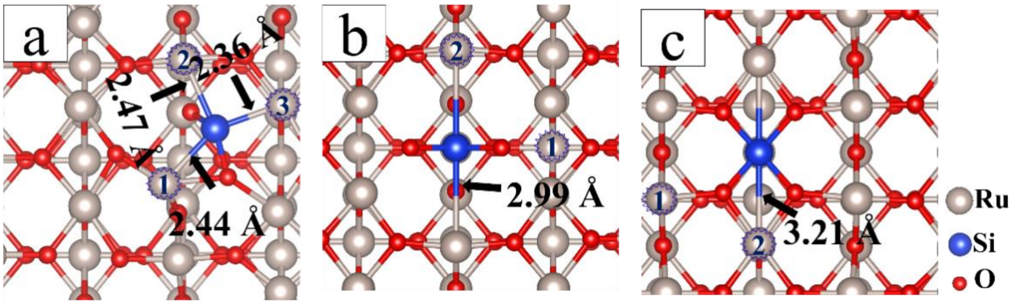

Figure S14. Si-RuO_x@C DFT calculation models. a) ①: Ru-intra-Si-1, ②: Ru-intra-Si-2 and ③: Ru-intra-Si. b) ①: Ru-O_5-Si and ②: Ru-O_5-Si-1. c) ①: Ru-O_4-Si and ②: Ru-O_4-Si-1.

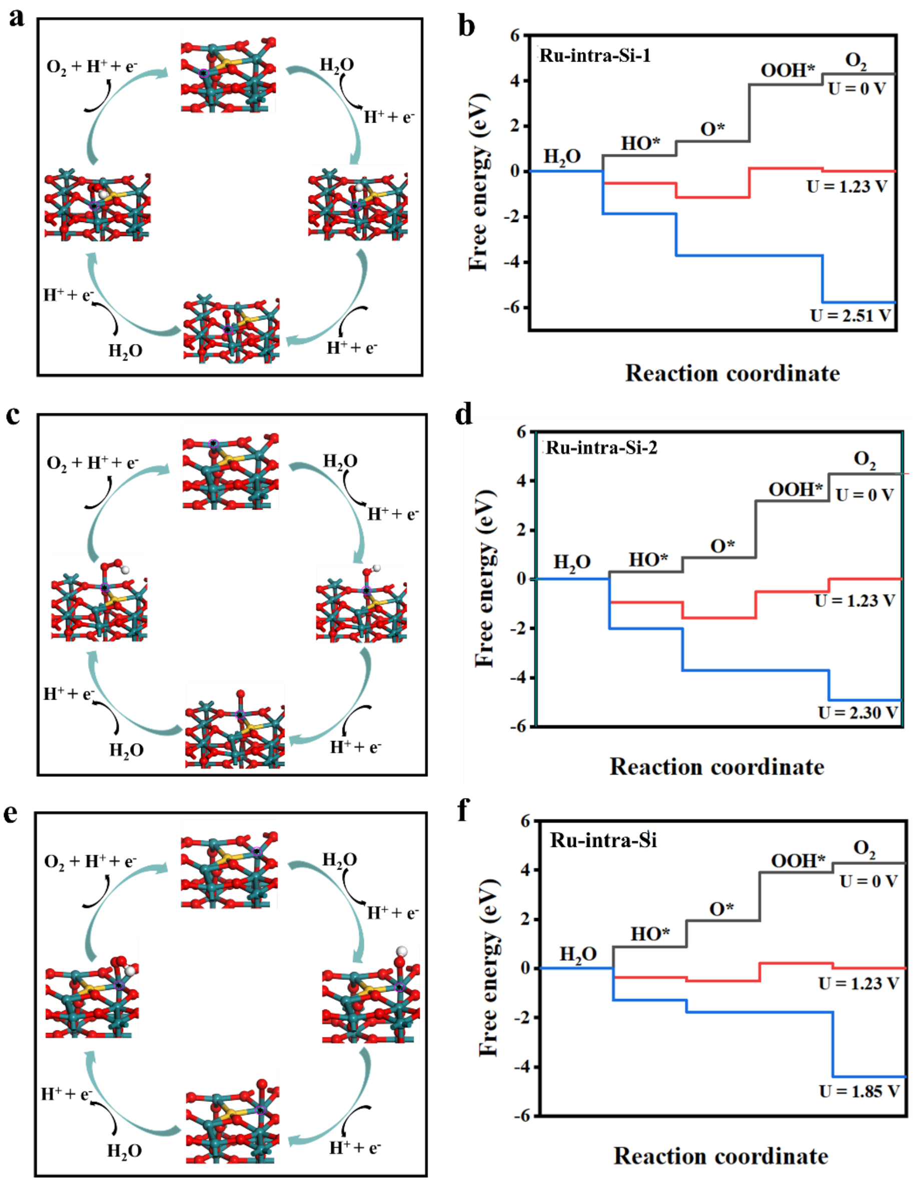

Figure S15. a), c) and e)The four-electron mechanism of Ru-intra-Si-1, Ru-intra-Si-2 and Ru-intra-Si toward acidic OER. b), d) and f) The Gibbs free energy diagrams for Ru-intra-Si-1, Ru-intra-Si-2 and Ru-intra-Si.

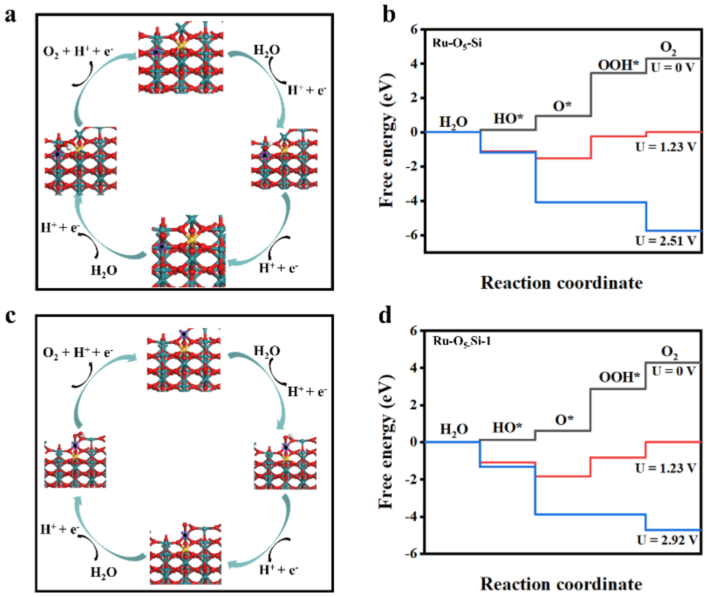

Figure S16. a) and c) The four-electron mechanism of $Ru-O_5-Si$ and $Ru-O_5-Si-1$ toward acidic OER. b) and d) The Gibbs free energy diagrams for $Ru-O_5-Si$ and $Ru-O_5-Si-1$.

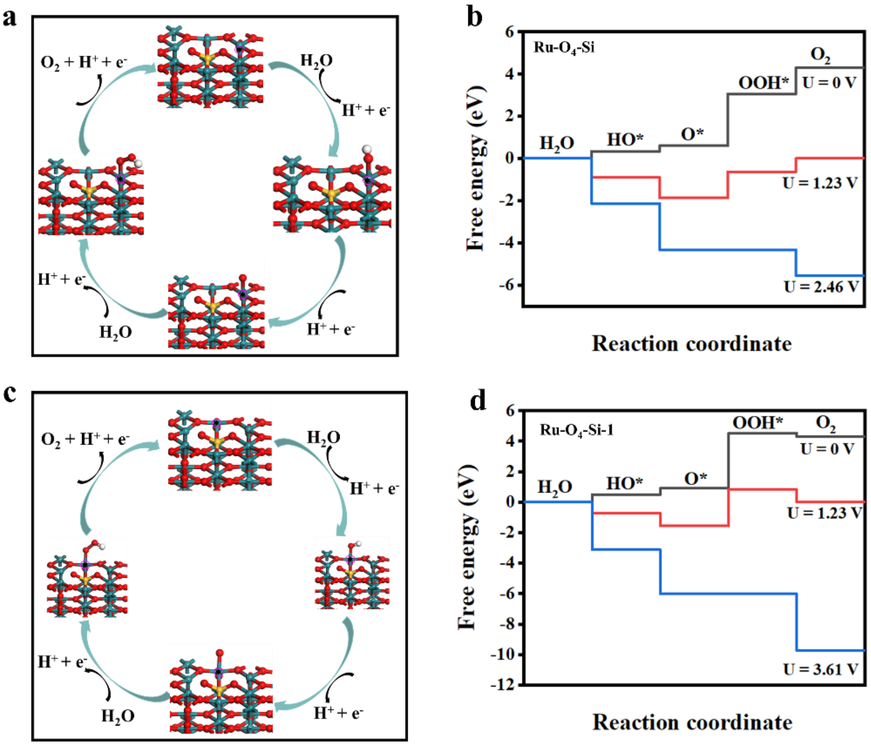

Figure S17. a) and c) The four-electron mechanism of $Ru-O_4-Si$ and $Ru-O_4-Si-1$ toward acidic OER. b) and d) The Gibbs free energy diagrams for $Ru-O_4-Si$ and $Ru-O_4-Si-1$.

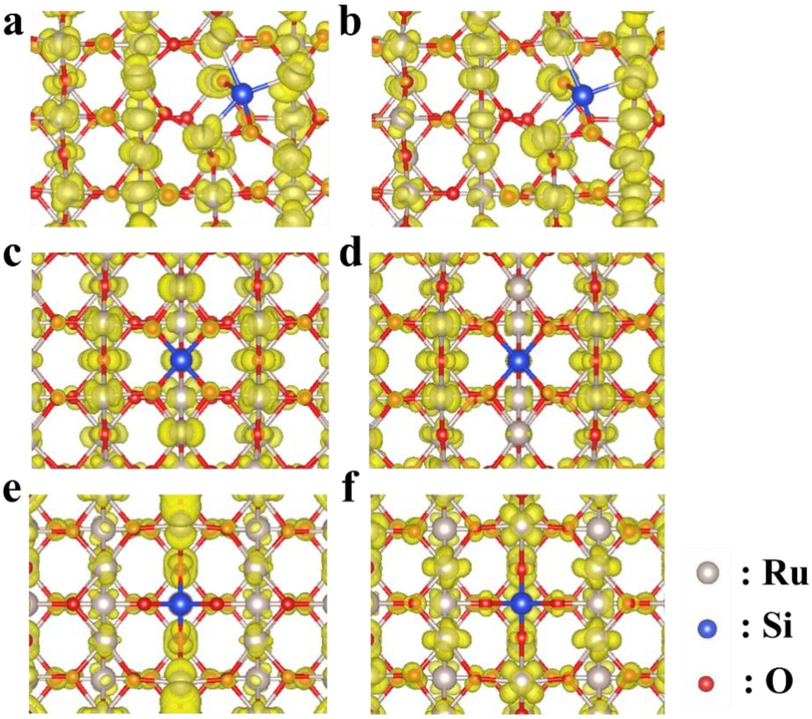

Figure S18. Si-RuO_x@C partial charge of different Si replacing Ru. a) and b) Ru-intra-Si. c) and d) Ru-O_5-Si. e) and f) Ru-O_4-Si.

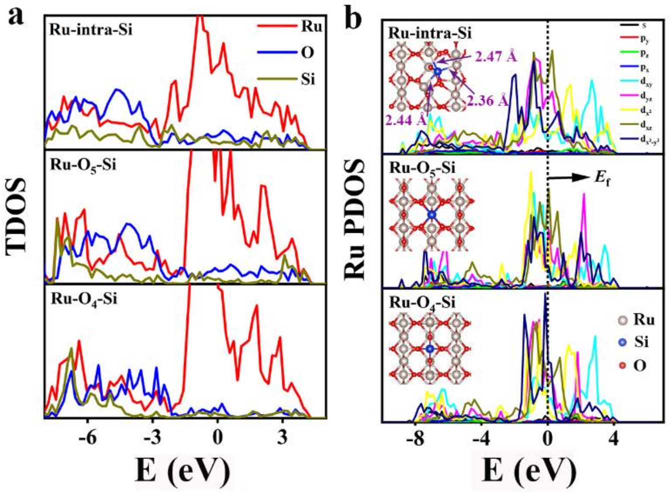

Figure S19. a) The TDOS of Ru-intra-Si, Ru-O_4-Si and Ru-O_5-Si. b) The PDOS of Ru for Ru-intra-Si, Ru-O_4-Si and Ru-O_5-Si.

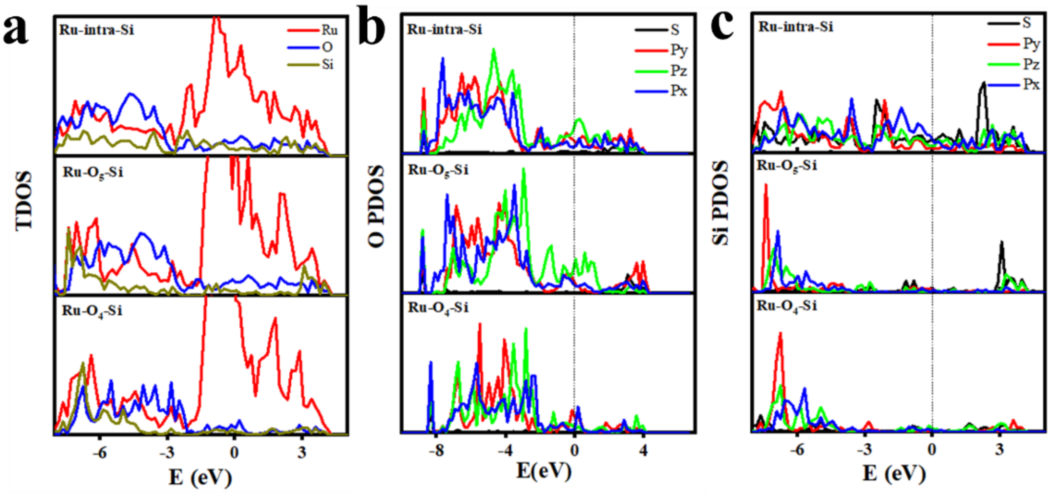

Figure S20. Ru-intra-Si, Ru-O_5-Si and Ru-O_4-Si of DOS. a) TDOS. b) The PDOS of O. c) The PDOS of Si.

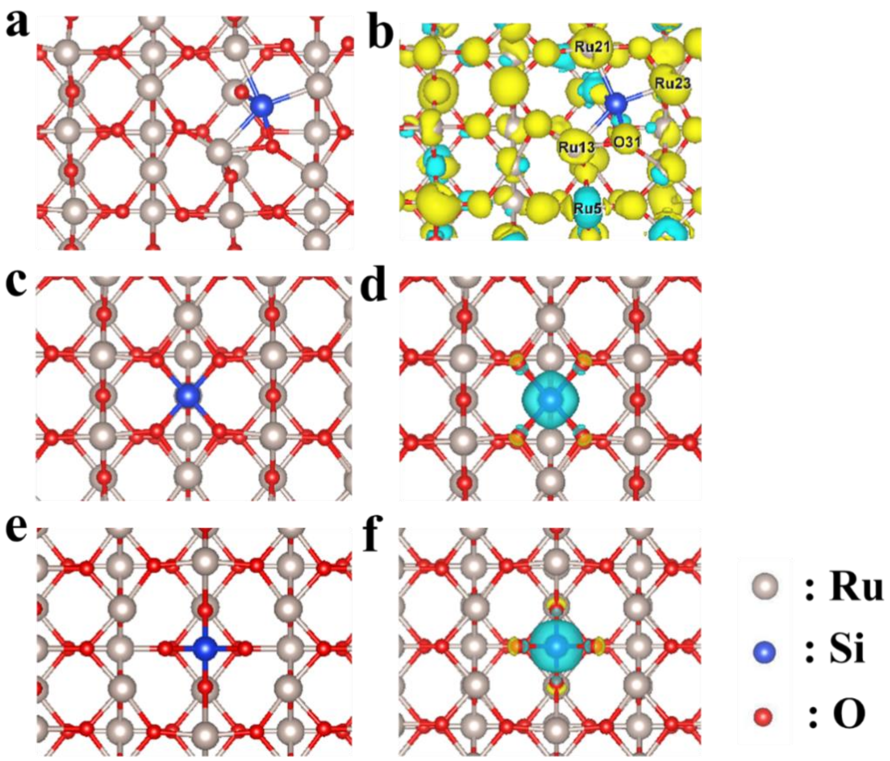

Figure S21. Differential charge density at the interface Ru centers between RuO_x and Si. Yellow and cyan contours represent electron accumulation and depletion, respectively. a) and b) Ru-intra-Si. c) and d) Ru-O_5-Si. e) and f) Ru-O_4-Si.

S3 Modeling

S3-1: Optimized structure information of Ru-intra-Si

```
 1.00000000000000
   12.7746000290000001    0.0000000000000000    0.0000000000000000
    0.0000000000000000    9.2770004272000008    0.0000000000000000
    0.0000000000000000    0.0000000000000000   16.9633998870999996
  Ru   O   Si
   24   48    1
Selective dynamics
Direct
  0.1342831815799521  0.1628312000135450  0.7818445823058006   T   T   T
  0.3735100099999968  0.1664700019999970  0.5962700369999965   F   F   F
  0.3722267396062425 -0.0102535843915347  0.7867733247599732   T   T   T
  0.1250000000000000  0.0000300000000024  0.5932999589999994   F   F   F
  0.6151273522371494  0.1543296480820611  0.7831056702361591   T   T   T
  0.8764899709999980  0.1664799990000034  0.5962700369999965   F   F   F
  0.8768372787994272  0.0348654914897338  0.7870651665594197   T   T   T
  0.6250000089999972  0.9970399810000004  0.5892799820000008   F   F   F
  0.1135891350840483  0.5068961112355548  0.7792799461987966   T   T   T
  0.3738000029999995  0.5004300120000025  0.5964099669999996   F   F   F
  0.3764651477502776  0.3420004936633695  0.7884725023699457   T   T   T
  0.1250000000000000  0.3334599909999980  0.5935599740000015   F   F   F
  0.5783068299798798  0.4207515394938743  0.7936355803733015   T   T   T
  0.8762000159999985  0.5004300120000025  0.5964099669999996   F   F   F
  0.8705870934837782  0.3209281259087828  0.7866336927374172   T   T   T
  0.6249599949999975  0.3483400140000015  0.5698599580000021   F   F   F
  0.1136180126109520  0.8412687926101394  0.7863901263718303   T   T   T
  0.3747499750000003  0.8332499970000029  0.5963500370000006   F   F   F
  0.3654959296133010  0.6813805820010193  0.7876636618756205   T   T   T
  0.1250000000000000  0.6664999939999987  0.5935500240000025   F   F   F
  0.6277478964790687  0.8581204785675300  0.7901914508122608   T   T   T
  0.8752499690000022  0.8332499970000029  0.5963399740000028   F   F   F
  0.8832933689654582  0.6964915506983015  0.7847080297677980   T   T   T
  0.6250100130000007  0.6693500169999993  0.5853199909999987   F   F   F
  0.1250000000000000  0.0000000000000000  0.4781000210000030   F   F   F
  0.3750199979999991  0.9999099469999990  0.5214400469999987   F   F   F
  0.3725681916073763 -0.0019345338929563  0.6745579088408045   T   T   T
  0.1215310853584145  0.0020868636469777  0.7110499172831852   T   T   T
  0.4652659481705139  0.1567255447361046  0.8007449385538519   T   T   T
  0.2225900010000004  0.1665599900000032  0.5956399849999983   F   F   F
  0.2813089392419789  0.1698165932469486  0.7991493694134381   T   T   T
  0.0274100020000034  0.1665599900000032  0.5956399849999983   F   F   F
  0.6250000089999972  0.0000000000000000  0.4781000210000030   F   F   F
  0.8749799460000034  0.9999099469999990  0.5214400469999987   F   F   F
  0.8789135636527921  0.0044358708909386  0.6747854604419632   T   T   T
  0.6226952913473185 -0.0030406160887296  0.7074019833986194   T   T   T
  0.9882991243337250  0.1812367865952139  0.8034747790062265   T   T   T
  0.7243999479999985  0.1631000040000004  0.5959699929999971   F   F   F
  0.7616261867693804  0.1673422933722294  0.8020370038709858   T   T   T
  0.5256100369999999  0.1631100020000034  0.5959699929999971   F   F   F
  0.1250000000000000  0.3333300010000002  0.4781000210000030   F   F   F
  0.3752099920000020  0.3333200040000008  0.5215099850000016   F   F   F
  0.3738208107310216  0.3335559614392560  0.6753457926175831   T   T   T
  0.1241602038022797  0.3365691804628109  0.7130770842450546   T   T   T
  0.4564313717483368  0.5206801581956382  0.8252791375378478   T   T   T
  0.2225600089999986  0.5000000000000000  0.5956299770000015   F   F   F
  0.2658107512145878  0.5099929793276344  0.8008396678637614   T   T   T
  0.0274400010000022  0.5000000000000000  0.5956299770000015   F   F   F
  0.6250000089999972  0.3333300010000002  0.4781000210000030   F   F   F
  0.8747900259999994  0.3333200040000008  0.5215099850000016   F   F   F
  0.8742808159432325  0.3323559572490103  0.6735688151838055   T   T   T
```

0.6147829650365318	0.3159609670978287	0.7052675702059231	T	T	T
0.9592665977767432	0.5049320428723399	0.7974772155147819	T	T	T
0.7248399590000005	0.5034899520000025	0.5953099760000029	F	F	F
0.7416804564833999	0.4447585761271655	0.8118095055024830	T	T	T
0.5251600229999980	0.5034899520000025	0.5953099760000029	F	F	F
0.1250000000000000	0.6666700250000019	0.4781000210000030	F	F	F
0.3749199989999994	0.6666999910000015	0.5214800199999985	F	F	F
0.3743285690969987	0.6679103437629353	0.6759802127540949	T	T	T
0.1256729053942614	0.6738288487007330	0.7120097496862310	T	T	T
0.4796754728314281	0.8375359694525135	0.8032528187741271	T	T	T
0.2226600070000018	0.8333699639999992	0.5956700059999989	F	F	F
0.2628742179436348	0.8356637695039987	0.8055809688895385	T	T	T
0.0273300010000028	0.8333699639999992	0.5956700059999989	F	F	F
0.6250000089999972	0.6666700250000019	0.4781000210000030	F	F	F
0.8750799820000026	0.6666999910000015	0.5214700120000018	F	F	F
0.8744831615105020	0.6667287207926200	0.6744544065788082	T	T	T
0.6417712702791911	0.6671708376484693	0.7086586086206157	T	T	T
0.9733707277346458	0.8713243114150064	0.8192275756671368	T	T	T
0.7227799579999967	0.8338199709999969	0.5952800109999998	F	F	F
0.7833586040433997	0.8679872665232095	0.7995540767002247	T	T	T
0.5272200240000018	0.8338299939999985	0.5952700039999996	F	F	F
0.7080078500632053	0.6147402138675931	0.7881257913253804	T	T	T

S3-2: Optimized structure information of Ru-O_5-Si

1.00000000000000
12.7746000290000001 0.0000000000000000 0.0000000000000000
0.0000000000000000 9.2770004272000008 0.0000000000000000
0.0000000000000000 0.0000000000000000 16.9633998870999996
Ru O Si
47 84 1
Selective dynamics
Direct

0.7517578884392704	0.3370715187757228	0.7790003421703239	T	T	T
0.2482420925607315	0.6629284552242750	0.7790003421703239	T	T	T
0.7517578884392704	0.6629284552242750	0.7790003421703239	T	T	T
0.2482420925607315	0.3370715187757228	0.7790003421703239	T	T	T
0.7499999810000020	0.3333300010000002	0.4037599839999970	F	F	F
0.2500000000000000	0.6666699729999976	0.4037599839999970	F	F	F
0.7499999810000020	0.6666699729999976	0.4037599839999970	F	F	F
0.2500000000000000	0.3333300010000002	0.4037599839999970	F	F	F
0.7499999810000020	0.1666699989999998	0.2170400090000015	F	F	F
0.2500000000000000	0.8333300270000024	0.2170400090000015	F	F	F
0.7499999810000020	0.8333300270000024	0.2170400090000015	F	F	F
0.2500000000000000	0.1666699989999998	0.2170400090000015	F	F	F
0.7497275688484313	0.1670578304175795	0.5926908230143353	T	T	T
0.2502724121515704	0.8329421955824224	0.5926908230143353	T	T	T
0.7497275688484313	0.8329421955824224	0.5926908230143353	T	T	T
0.2502724121515704	0.1670578304175795	0.5926908230143353	T	T	T
0.0000000000000000	0.3333300010000002	0.2218700119999966	F	F	F
0.0000000000000000	0.6666699729999976	0.2218700119999966	F	F	F
-0.0000000000000000	0.3338396095315111	0.5971997258638047	T	T	T
-0.0000000000000000	0.6661603644684867	0.5971997258638047	T	T	T
-0.0000000000000000	0.1485520226106694	0.7833149515707718	T	T	T
-0.0000000000000000	0.8514480033893328	0.7833149515707718	T	T	T
0.0000000000000000	0.1666699989999998	0.4069599999999980	F	F	F
0.0000000000000000	0.8333300270000024	0.4069599999999980	F	F	F
0.5000000000000000	0.3333300010000002	0.2218700119999966	F	F	F
0.5000000000000000	0.6666699729999976	0.2218700119999966	F	F	F
0.5000000000000000	0.3321299875528147	0.5945801371658349	T	T	T
0.5000000000000000	0.6678699864471833	0.5945801371658349	T	T	T
0.5000000000000000	0.1774443916022496	0.7819821445056887	T	T	T
0.5000000000000000	0.8225556343977524	0.7819821445056887	T	T	T
0.5000000000000000	0.1666699989999998	0.4069599999999980	F	F	F

0.5000000000000000	0.8333300270000024	0.4069599999999980	F	F	F
0.7499999810000020	0.5000000000000000	0.2170400090000015	F	F	F
0.2500000000000000	0.5000000000000000	0.2170400090000015	F	F	F
0.7498806792795764	0.5000000000000000	0.5925997043414044	T	T	T
0.2501193017204256	0.5000000000000000	0.5925997043414044	T	T	T
-0.0000000000000000	0.5000000000000000	0.7858434692954439	T	T	T
0.0000000000000000	0.5000000000000000	0.4069599999999980	F	F	F
0.5000000000000000	0.5000000000000000	0.4069599999999980	F	F	F
0.0000000000000000	0.0000000000000000	0.2218700119999966	F	F	F
-0.0000000000000000	-0.0000000000000000	0.5953726619509483	T	T	T
0.7399658709799455	-0.0000000000000000	0.7785449988207538	T	T	T
0.2600341100200566	-0.0000000000000000	0.7785449988207538	T	T	T
0.7499999810000020	0.0000000000000000	0.4037599839999970	F	F	F
0.2500000000000000	0.0000000000000000	0.4037599839999970	F	F	F
0.5000000000000000	0.0000000000000000	0.2218700119999966	F	F	F
0.5000000000000000	-0.0000000000000000	0.5964511768759505	T	T	T
0.7499999810000020	0.1666699989999998	0.4781000210000030	F	F	F
0.2500000000000000	0.8333300270000024	0.4781000210000030	F	F	F
0.7499999810000020	0.8333300270000024	0.4781000210000030	F	F	F
0.2500000000000000	0.1666699989999998	0.4781000210000030	F	F	F
0.7475469982640663	0.1693056922398953	0.7076306977255792	T	T	T
0.2524529827359355	0.8306943337601069	0.7076306977255792	T	T	T
0.7475469982640663	0.8306943337601069	0.7076306977255792	T	T	T
0.2524529827359355	0.1693056922398953	0.7076306977255792	T	T	T
0.7499999810000020	0.1666699989999998	0.3294300120000031	F	F	F
0.2500000000000000	0.8333300270000024	0.3294300120000031	F	F	F
0.7499999810000020	0.8333300270000024	0.3294300120000031	F	F	F
0.2500000000000000	0.1666699989999998	0.3294300120000031	F	F	F
0.6496900109999970	0.3333300010000002	0.1999399970000013	F	F	F
0.3503099890000030	0.6666699729999976	0.1999399970000013	F	F	F
0.6496900109999970	0.6666699729999976	0.1999399970000013	F	F	F
0.3503099890000030	0.3333300010000002	0.1999399970000013	F	F	F
0.9026200469999992	0.3333300010000002	0.4038000130000015	F	F	F
0.0973799989999975	0.6666699729999976	0.4038000130000015	F	F	F
0.9026200469999992	0.6666699729999976	0.4038000130000015	F	F	F
0.0973799989999975	0.3333300010000002	0.4038000130000015	F	F	F
0.8503099520000035	0.3333300010000002	0.1999399970000013	F	F	F
0.1496900010000033	0.6666699729999976	0.1999399970000013	F	F	F
0.8503099520000035	0.6666699729999976	0.1999399970000013	F	F	F
0.1496900010000033	0.3333300010000002	0.1999399970000013	F	F	F
0.0976674314199376	0.3276202029982748	0.7952759605276652	T	T	T
0.9023325965800573	0.6723797710017227	0.7952759605276652	T	T	T
0.0976674314199376	0.6723797710017227	0.7952759605276652	T	T	T
0.9023325965800573	0.3276202029982748	0.7952759605276652	T	T	T
0.8477692880823441	0.3333774200473976	0.5942538258308109	T	T	T
0.1522307219176567	0.6666225539526001	0.5942538258308109	T	T	T
0.8477692880823441	0.6666225539526001	0.5942538258308109	T	T	T
0.1522307219176567	0.3333774200473976	0.5942538258308109	T	T	T
0.6515024576415435	0.3337327900345408	0.5925437536630022	T	T	T
0.3484975053584570	0.6662671839654573	0.5925437536630022	T	T	T
0.6515024576415435	0.6662671839654573	0.5925437536630022	T	T	T
0.3484975053584570	0.3337327900345408	0.5925437536630022	T	T	T
0.4026199730000002	0.3333300010000002	0.4038000130000015	F	F	F
0.5973800269999998	0.6666699729999976	0.4038000130000015	F	F	F
0.4026199730000002	0.6666699729999976	0.4038000130000015	F	F	F
0.5973800269999998	0.3333300010000002	0.4038000130000015	F	F	F
0.5924298979933527	0.3573329926029064	0.7930339695772228	T	T	T
0.4075701020066476	0.6426669813970916	0.7930339695772228	T	T	T
0.5924298979933527	0.6426669813970916	0.7930339695772228	T	T	T
0.4075701020066476	0.3573329926029064	0.7930339695772228	T	T	T
-0.0000000000000000	0.1676280167416447	0.5213295915977543	T	T	T
-0.0000000000000000	0.8323720092583574	0.5213295915977543	T	T	T
0.0000000000000000	0.1666699989999998	0.2897300019999989	F	F	F
0.0000000000000000	0.8333300270000024	0.2897300019999989	F	F	F
-0.0000000000000000	0.1651042207749199	0.6695628396366933	T	T	T
-0.0000000000000000	0.8348958052250823	0.6695628396366933	T	T	T
0.5000000000000000	0.1665159013736313	0.5210515827438966	T	T	T

0.5000000000000000	0.8334841246263709	0.5210515827438966	T	T	T
0.5000000000000000	0.1666699989999998	0.2897300019999989	F	F	F
0.5000000000000000	0.8333300270000024	0.2897300019999989	F	F	F
0.5000000000000000	0.1675940650026730	0.6693126168676233	T	T	T
0.5000000000000000	0.8324059609973292	0.6693126168676233	T	T	T
0.7499999810000020	0.5000000000000000	0.4781000210000030	F	F	F
0.2500000000000000	0.5000000000000000	0.4781000210000030	F	F	F
0.7505498125503971	0.5000000000000000	0.7060986351417008	T	T	T
0.2494501684496051	0.5000000000000000	0.7060986351417008	T	T	T
0.7499999810000020	0.5000000000000000	0.3294300120000031	F	F	F
0.2500000000000000	0.5000000000000000	0.3294300120000031	F	F	F
-0.0000000000000000	0.5000000000000000	0.5216275286663410	T	T	T
0.0000000000000000	0.5000000000000000	0.2897300019999989	F	F	F
-0.0000000000000000	0.5000000000000000	0.6723543007363273	T	T	T
0.5000000000000000	0.5000000000000000	0.5207804307720092	T	T	T
0.5000000000000000	0.5000000000000000	0.2897300019999989	F	F	F
0.5000000000000000	0.5000000000000000	0.6730902711559907	T	T	T
0.6496900109999970	0.0000000000000000	0.1999399970000013	F	F	F
0.3503099890000030	0.0000000000000000	0.1999399970000013	F	F	F
0.9026200469999992	0.0000000000000000	0.4038000130000015	F	F	F
0.0973799989999975	0.0000000000000000	0.4038000130000015	F	F	F
0.8503099520000035	0.0000000000000000	0.1999399970000013	F	F	F
0.1496900010000033	0.0000000000000000	0.1999399970000013	F	F	F
0.1123211844455559	-0.0000000000000000	0.7978848781477749	T	T	T
0.8876788435544393	-0.0000000000000000	0.7978848781477749	T	T	T
0.8474262396188776	-0.0000000000000000	0.5936588805486446	T	T	T
0.1525737703811232	-0.0000000000000000	0.5936588805486446	T	T	T
0.6527290171771454	-0.0000000000000000	0.5937790945835016	T	T	T
0.3472709458228549	-0.0000000000000000	0.5937790945835016	T	T	T
0.4026199730000002	0.0000000000000000	0.4038000130000015	F	F	F
0.5973800269999998	0.0000000000000000	0.4038000130000015	F	F	F
0.5925869279306872	-0.0000000000000000	0.7894511713617683	T	T	T
0.4074130720693127	-0.0000000000000000	0.7894511713617683	T	T	T
0.5000000000000000	0.5000000000000000	0.7714616566735302	T	T	T

S3-3: Optimized structure information of Ru-O_4-Si

1.00000000000000					
12.7746000290000001	0.0000000000000000	0.0000000000000000			
0.0000000000000000	9.2770004272000008	0.0000000000000000			
0.0000000000000000	0.0000000000000000	16.9633998870999996			
Ru	O	Si			
47	84	1			
Selective dynamics					
Direct					
0.2500000000000000	0.1666699989999998	0.2218700119999966	F	F	F
0.7499999810000020	0.8333300270000024	0.2218700119999966	F	F	F
0.2500000000000000	0.8333300270000024	0.2218700119999966	F	F	F
0.7499999810000020	0.1666699989999998	0.2218700119999966	F	F	F
0.2499568608285129	0.1669259042513238	0.5972289587431097	T	T	T
0.7500431201714891	0.8330741217486783	0.5972289587431097	T	T	T
0.2499568608285129	0.8330741217486783	0.5972289587431097	T	T	T
0.7500431201714891	0.1669259042513238	0.5972289587431097	T	T	T
0.2566621685951537	0.3390863227773888	0.7837982009170242	T	T	T
0.7433378124048484	0.6609136512226090	0.7837982009170242	T	T	T
0.2566621685951537	0.6609136512226090	0.7837982009170242	T	T	T
0.7433378124048484	0.3390863227773888	0.7837982009170242	T	T	T
0.2500000000000000	0.3333300010000002	0.4069599999999980	F	F	F
0.7499999810000020	0.6666699729999976	0.4069599999999980	F	F	F
0.2500000000000000	0.6666699729999976	0.4069599999999980	F	F	F
0.7499999810000020	0.3333300010000002	0.4069599999999980	F	F	F
0.0000000000000000	0.1757037605856138	0.7764364290733806	T	T	T
0.0000000000000000	0.8242962654143884	0.7764364290733806	T	T	T
0.0000000000000000	0.1666699989999998	0.4037599839999970	F	F	F

0.0000000000000000	0.8333300270000024	0.4037599839999970	F	F	F
0.0000000000000000	0.3333300010000002	0.2170400090000015	F	F	F
0.0000000000000000	0.6666699729999976	0.2170400090000015	F	F	F
0.0000000000000000	0.3342104419386361	0.5919421655950339	T	T	T
0.0000000000000000	0.6657895320613618	0.5919421655950339	T	T	T
0.0000000000000000	0.0000000000000000	0.2170400090000015	F	F	F
0.0000000000000000	-0.0000000000000000	0.5932469272328562	T	T	T
0.2480767284056249	-0.0000000000000000	0.7831120674965126	T	T	T
0.7519232525943771	-0.0000000000000000	0.7831120674965126	T	T	T
0.2500000000000000	0.0000000000000000	0.4069599999999980	F	F	F
0.7499999810000020	0.0000000000000000	0.4069599999999980	F	F	F
0.5000000000000000	0.1483134486312015	0.7811361306326695	T	T	T
0.5000000000000000	0.8516865773688007	0.7811361306326695	T	T	T
0.5000000000000000	0.1666699989999998	0.4037599839999970	F	F	F
0.5000000000000000	0.8333300270000024	0.4037599839999970	F	F	F
0.5000000000000000	0.3333300010000002	0.2170400090000015	F	F	F
0.5000000000000000	0.6666699729999976	0.2170400090000015	F	F	F
0.5000000000000000	0.3336248670415082	0.5906561618012344	T	T	T
0.5000000000000000	0.6663751069584896	0.5906561618012344	T	T	T
0.5000000000000000	0.0000000000000000	0.2170400090000015	F	F	F
0.5000000000000000	-0.0000000000000000	0.5910102827566550	T	T	T
0.2500000000000000	0.5000000000000000	0.2218700119999966	F	F	F
0.7499999810000020	0.5000000000000000	0.2218700119999966	F	F	F
0.2507784450102363	0.5000000000000000	0.5967954804433477	T	T	T
0.7492215359897655	0.5000000000000000	0.5967954804433477	T	T	T
0.0000000000000000	0.5000000000000000	0.7817506355009595	T	T	T
0.0000000000000000	0.5000000000000000	0.4037599839999970	F	F	F
0.5000000000000000	0.5000000000000000	0.4037599839999970	F	F	F
0.8996900289999985	0.1666699989999998	0.1999399970000013	F	F	F
0.1003099989999967	0.8333300270000024	0.1999399970000013	F	F	F
0.8996900289999985	0.8333300270000024	0.1999399970000013	F	F	F
0.1003099989999967	0.1666699989999998	0.1999399970000013	F	F	F
0.1526200010000025	0.1666699989999998	0.4038000130000015	F	F	F
0.8473800089999983	0.8333300270000024	0.4038000130000015	F	F	F
0.1526200010000025	0.8333300270000024	0.4038000130000015	F	F	F
0.8473800089999983	0.1666699989999998	0.4038000130000015	F	F	F
0.3473800089999983	0.1666699989999998	0.4038000130000015	F	F	F
0.6526199540000022	0.8333300270000024	0.4038000130000015	F	F	F
0.3473800089999983	0.8333300270000024	0.4038000130000015	F	F	F
0.6526199540000022	0.1666699989999998	0.4038000130000015	F	F	F
0.3494724859699377	0.1644762000100082	0.7931391718475365	T	T	T
0.6505275140300625	0.8355238259899940	0.7931391718475365	T	T	T
0.3494724859699377	0.8355238259899940	0.7931391718475365	T	T	T
0.6505275140300625	0.1644762000100082	0.7931391718475365	T	T	T
0.0970182974761395	0.1670096773419704	0.5948030866229357	T	T	T
0.9029817485238573	0.8329903486580318	0.5948030866229357	T	T	T
0.0970182974761395	0.8329903486580318	0.5948030866229357	T	T	T
0.9029817485238573	0.1670096773419704	0.5948030866229357	T	T	T
0.1520984211523480	0.1771689652103799	0.7906133315693840	T	T	T
0.8479015318476585	0.8228310607896224	0.7906133315693840	T	T	T
0.1520984211523480	0.8228310607896224	0.7906133315693840	T	T	T
0.8479015318476585	0.1771689652103799	0.7906133315693840	T	T	T
0.3996899919999990	0.1666699989999998	0.1999399970000013	F	F	F
0.6003099710000015	0.8333300270000024	0.1999399970000013	F	F	F
0.3996899919999990	0.8333300270000024	0.1999399970000013	F	F	F
0.6003099710000015	0.1666699989999998	0.1999399970000013	F	F	F
0.5969609365541597	0.1679439654070300	0.5945437011316416	T	T	T
0.4030390634458403	0.8320560605929721	0.5945437011316416	T	T	T
0.5969609365541597	0.8320560605929721	0.5945437011316416	T	T	T
0.4030390634458403	0.1679439654070300	0.5945437011316416	T	T	T
0.2498318710175752	0.3332461746976630	0.5217788612501877	T	T	T
0.7501681099824268	0.6667537993023349	0.5217788612501877	T	T	T
0.2498318710175752	0.6667537993023349	0.5217788612501877	T	T	T
0.7501681099824268	0.3332461746976630	0.5217788612501877	T	T	T
0.2500000000000000	0.3333300010000002	0.2897300019999989	F	F	F
0.7499999810000020	0.6666699729999976	0.2897300019999989	F	F	F
0.2500000000000000	0.6666699729999976	0.2897300019999989	F	F	F

0.7499999810000020	0.3333300010000002	0.2897300019999989	F	F	F
0.2520983765308236	0.3337346535338390	0.6705706034786839	T	T	T
0.7479016044691783	0.6662653204661587	0.6705706034786839	T	T	T
0.2520983765308236	0.6662653204661587	0.6705706034786839	T	T	T
0.7479016044691783	0.3337346535338390	0.6705706034786839	T	T	T
0.0000000000000000	0.3333300010000002	0.4781000210000030	F	F	F
0.0000000000000000	0.6666699729999976	0.4781000210000030	F	F	F
0.0000000000000000	0.3386109315502260	0.7078760861548659	T	T	T
0.0000000000000000	0.6613890424497719	0.7078760861548659	T	T	T
0.0000000000000000	0.3333300010000002	0.3294300120000031	F	F	F
0.0000000000000000	0.6666699729999976	0.3294300120000031	F	F	F
0.0000000000000000	0.0000000000000000	0.4781000210000030	F	F	F
0.0000000000000000	-0.0000000000000000	0.7109396910077961	T	T	T
0.0000000000000000	0.0000000000000000	0.3294300120000031	F	F	F
0.2498341775782878	-0.0000000000000000	0.5220623287819242	T	T	T
0.7501658034217145	-0.0000000000000000	0.5220623287819242	T	T	T
0.2500000000000000	0.0000000000000000	0.2897300019999989	F	F	F
0.7499999810000020	0.0000000000000000	0.2897300019999989	F	F	F
0.2493650306382748	-0.0000000000000000	0.6707229445950591	T	T	T
0.7506349503617269	-0.0000000000000000	0.6707229445950591	T	T	T
0.5000000000000000	0.3333300010000002	0.4781000210000030	F	F	F
0.5000000000000000	0.6666699729999976	0.4781000210000030	F	F	F
0.5000000000000000	0.3458738540322523	0.7110433668945840	T	T	T
0.5000000000000000	0.6541261199677453	0.7110433668945840	T	T	T
0.5000000000000000	0.3333300010000002	0.3294300120000031	F	F	F
0.5000000000000000	0.6666699729999976	0.3294300120000031	F	F	F
0.5000000000000000	0.0000000000000000	0.4781000210000030	F	F	F
0.5000000000000000	-0.0000000000000000	0.7067778534002052	T	T	T
0.5000000000000000	0.0000000000000000	0.3294300120000031	F	F	F
0.8996900289999985	0.5000000000000000	0.1999399970000013	F	F	F
0.1003099989999967	0.5000000000000000	0.1999399970000013	F	F	F
0.1526200010000025	0.5000000000000000	0.4038000130000015	F	F	F
0.8473800089999983	0.5000000000000000	0.4038000130000015	F	F	F
0.3473800089999983	0.5000000000000000	0.4038000130000015	F	F	F
0.6526199540000022	0.5000000000000000	0.4038000130000015	F	F	F
0.3827110650602688	0.5000000000000000	0.8059828387766678	T	T	T
0.6172889349397311	0.5000000000000000	0.8059828387766678	T	T	T
0.0979207049270410	0.5000000000000000	0.5937734116759990	T	T	T
0.9020793410729556	0.5000000000000000	0.5937734116759990	T	T	T
0.1528865247295780	0.5000000000000000	0.7957939359747010	T	T	T
0.8471134282704289	0.5000000000000000	0.7957939359747010	T	T	T
0.3996899919999990	0.5000000000000000	0.1999399970000013	F	F	F
0.6003099710000015	0.5000000000000000	0.1999399970000013	F	F	F
0.5972112047469186	0.5000000000000000	0.5922958606449015	T	T	T
0.4027887952530814	0.5000000000000000	0.5922958606449015	T	T	T
0.5000000000000000	0.5000000000000000	0.7600514605517775	T	T	T

REFERENCES

[1] G. Pan, C. Hu, S. Hong, H. Li, D. Yu, C. Cui, Q. Li, N. Liang, Y. Jiang, L. Zheng, L. Jiang, Y. Liu, *Nat. Commun.* **2021**, *12*, 64.

[2] G. Kresse, J. Hafner, *Phys. Rev. B.* **1993**, *47*, 558.

[3] G. Kresse, J. Hafner, *Phys. Rev. B.* **1994**, *49*, 14251.

[4] G. Kresse, J. Furthmuller, *Comp. Mater. Sci.* **1996**, *6*, 15.

[5] G. Kresse, J. Furthmuller, *Phys. Rev. B.* **1996**, *54*, 11169.

[6] D. Vanderbilt, *Phys. Rev. B: Condens, Matter Mater. Phys*. **1990**, *41*, 7892.

[7] B. Hammer, L. B. Hansen and J. K. Norskov, *Phys. Rev. B: Condens. Matter Mater. Phys.* **1999**, *59*, 7413.

[8] P. E. Blochl, *Phys. Rev. B.* **1994**, *50*, 17953.

[9] G. Kresse, D. Joubert, *Phys. Rev. B.* **1999**, *59*, 1758.

[10] S. J. Grimme, *Comput. Chem.* **2004**, *25*, 1463.

[11] L. Yang, G. Yu, X. Ai, W. Yan, H. Duan, W. Chen, X. Li, T. Wang, C. Zhang, X. Huang, J. S. Chen, X. Zou, *Nat. Commun.* **2018**, *9*, 5236.

[12] J. Chen, P. Cui, G. Zhao, K. Rui, M. Lao, Y. Chen, X. Zheng, Y. Jiang, H. Pan, S. X. Dou, W. Sun, *Angew. Chem. Int. Ed.* **2019**, *58*, 12540.

[13] J. Shan, C. Guo, Y. Zhu, S. Chen, L. Song, M. Jaroniec, Y. Zheng, S. Qiao, *Chem* **2019**, *5*, 445.
[14] Y. Pi, Q. Shao, P. Wang, J. Guo, X. Huang, *Adv. Funct. Mater.* **2017**, *27*, 1700886.
[15] J. Gao, C. Xu, S. Hung, W. Liu, W. Cai, Z. Zeng, C. Jia, H. Chen, H. Xiao, J. Li, Y. Huang, B. Liu, *J. Am. Chem. Soc.* **2019**, *141*, 3014.
[16] R. Gao, Q. Zhang, H. Chen, X. Chu, G. Li, X. Zou, *J. Energy Chem.* **2020**, *47*, 291.
[17] G. Li, S. Li, J. Ge, C. Liu, W. Xing, *J. Mater. Chem. A* **2017**, *5*, 17221.
[18] T. Kwon, H. Hwang, Y. J. Sa, J. Park, H. Baik, S. H. Joo, K. Lee, *Adv. Funct. Mater.* **2017**, *27*, 1604688.
[19] S. Kumari, B. P. Ajayi, B. Kumar, J. B. Jasinski, M. K. Sunkara, J. M. Spurgeon, *Energy Environ. Sci.* **2017**, *10*, 2432.
[20] R. Li, H. Wang, F. Hu, K. Chan, X. Liu, Z. Lu, J. Wang, Z. Li, L. Zeng, Y. Li, X. Wu, Y. Xiong, *Nat. Commun.* **2021**, *12*, 3540.
[21] L. C. Seitz, C. F. Dickens, K. Nishio, Y. Hikita, J. Montoya, A. Doyle, C. Kirk, A. Vojvodic, H. Y. Hwang, J. K. Norskov, T. F. Jaramillo, *Science* **2016**, *353*, 6303.
[22] G. C. da Silva, M. R. Fernandes, E. A. Ticianelli, *ACS Catal.* **2018**, *8*, 2081.
[23] Q. Shi, C. Zhu, H. Zhong, D. Su, N. Li, M. H. Engelhard, H. Xia, Q. Zhang, S. Feng, S. P. Beckman, D. Du, Y. Lin, *ACS Energy Lett.* **2018**, *3*, 2038.
[24] J. Zhu, M. Xie, Z. Chen, Z. Lyu, M. Chi, W. Jin, Y. Xia, *Adv. Energy Mater.* **2020**, *10*, 1904114.
[25] P. Lettenmeier, L. Wang, U. Golla-Schindler, P. Gazdzicki, N. A. Canas, M. Handl, R. Hiesgen, S. S. Hosseiny, A. S. Gago, K. A. Friedrich, *Angew. Chem. Int. Ed.* **2016**, *55*, 742.
